# Tailored Modulation of Cellular Pro-inflammatory Responses With Disaccharide Lipid A Mimetics

**DOI:** 10.3389/fimmu.2021.631797

**Published:** 2021-03-18

**Authors:** Holger Heine, Florian Adanitsch, Tina Tinkara Peternelj, Mira Haegman, Christoph Kasper, Simon Ittig, Rudi Beyaert, Roman Jerala, Alla Zamyatina

**Affiliations:** ^1^Research Group Innate Immunity, Research Center Borstel - Leibniz Lung Center, Airway Research Center North (ARCN), German Center for Lung Research (DZL), Borstel, Germany; ^2^Department of Chemistry, University of Natural Resources and Life Sciences, Vienna, Austria; ^3^Department of Biotechnology, National Institute of Chemistry, University of Ljubljana, Ljubljana, Slovenia; ^4^Unit of Molecular Signal Transduction in Inflammation, Department of Biomedical Molecular Biology, Ghent University, Center for Inflammation Research, VIB, Ghent, Belgium; ^5^Biozentrum University of Basel, Basel, Switzerland

**Keywords:** synthetic TLR4 agonist, immunomodulation, lipopolysaccharide, Toll-like Receptor 4, TLR4/MD-2 complex activation, potential adjuvant, immune cells activation, innate immunity

## Abstract

Pro-inflammatory signaling mediated by Toll-like receptor 4 (TLR4)/myeloid differentiation-2 (MD-2) complex plays a crucial role in the instantaneous protection against infectious challenge and largely contributes to recovery from Gram-negative infection. Activation of TLR4 also boosts the adaptive immunity which is implemented in the development of vaccine adjuvants by application of minimally toxic TLR4 activating ligands. The modulation of pro-inflammatory responses via the TLR4 signaling pathway was found beneficial for management of acute and chronic inflammatory disorders including asthma, allergy, arthritis, Alzheimer disease pathology, sepsis, and cancer. The TLR4/MD-2 complex can recognize the terminal motif of Gram-negative bacterial lipopolysaccharide (LPS)—a glycophospholipid lipid A. Although immense progress in understanding the molecular basis of LPS-induced TLR4-mediated signaling has been achieved, gradual, and predictable TLR4 activation by structurally defined ligands has not yet been attained. We report on controllable modulation of cellular pro-inflammatory responses by application of novel synthetic glycolipids—disaccharide-based lipid A mimetics (DLAMs) having picomolar affinity for TLR4/MD-2. Using crystal structure inspired design we have developed endotoxin mimetics where the inherently flexible β(1 → 6)-linked diglucosamine backbone of lipid A is replaced by a conformationally restricted α,α-(1↔1)-linked disaccharide scaffold. The tertiary structure of the disaccharide skeleton of DLAMs mirrors the 3-dimensional shape of TLR4/MD-2 bound *E. coli* lipid A. Due to exceptional conformational rigidity of the sugar scaffold, the specific 3D organization of DLAM must be preserved upon interaction with proteins. These structural factors along with specific acylation and phosphorylation pattern can ensure picomolar affinity for TLR4 and permit efficient dimerization of TLR4/MD-2/DLAM complexes. Since the binding pose of lipid A in the binding pocket of MD-2 (±180°) is crucial for the expression of biological activity, the chemical structure of DLAMs was designed to permit a predefined binding orientation in the binding groove of MD-2, which ensured tailored and species-independent (human and mice) TLR4 activation. Manipulating phosphorylation and acylation pattern at the sugar moiety facing the secondary dimerization interface allowed for adjustable modulation of the TLR4-mediated signaling. Tailored modulation of cellular pro-inflammatory responses by distinct modifications of the molecular structure of DLAMs was attained in primary human and mouse immune cells, lung epithelial cells and TLR4 transfected HEK293 cells.

## Introduction

The instantaneous immune response to infection by Gram-negative bacteria depends on the structure of lipopolysaccharide (LPS, also branded as Endotoxin), a large (MW ca. 20 kDa) complex heterogeneous glycan constituting the outer leaflet of the bacterial outer membrane (1). A small amphiphilic terminal motif of LPS known as lipid A is responsible for induction of the host innate immune responses by interacting with several pattern recognition receptors (PRRs) normally expressed by mammalian immune cells. Detection of picomolar quantities of pathogenic LPS by a particular host PRR—the germline-encoded transmembrane Toll-like Receptor 4 (TLR4) complex—results in the initiation of diverse intracellular pro-inflammatory signaling cascades (2). An acute inflammatory response triggered upon activation of TLR4 by endotoxic lipid A involves downstream expression of pro-inflammatory cytokines, adhesive proteins, prostaglandins, and reactive oxygen species and is directed at elimination of bacterial disease (3). Thus, activation of the innate immune response through LPS-driven engagement of TLR4 during infection inherently contributes to spontaneous healing in immunocompetent host. However, uncontrolled upregulation of TLR4—mediated signaling can lead to an unrestrained cytokine storm and sepsis which provoked the development of TLR4 inhibiting strategies (4–6). On the other side, it has been shown that the inefficiency of the TLR4-driven inflammation could be responsible for the pathogenicity of Gram-negative sepsis (7–9). Besides, activation of TLR4 links the innate and adaptive immunity (10, 11) justifying application of less toxic TLR4 agonists as vaccine adjuvants (12, 13). The pivotal “endotoxic principle” of LPS resides in its rather small (MW ca. 2 kDa) terminal amphiphilic motif—a glycophospholipid lipid A which can be directly bound by the hydrophobic binding pocket of myeloid differentiation factor 2 (MD-2)—a TLR4 co-receptor protein (14). Binding of lipid A provokes dimerization of two TLR4/MD-2/LPS complexes which results in the activation of adaptor proteins and formation of a MyDDosome—an intracellular supramolecular organizing centre (SMOCs) coordinating the induction of the pro-inflammatory signaling cascades (2, 15–17). LPS-induced assembly of the dimeric [TLR4/MD-2/LPS]_2_ complexes triggers conformational changes in the intracellular Toll/IL-1R (TIR) domain of TLR4 (18). The latter event leads to the activation of diverse signaling molecules: MyD88 (myeloid differentiation primary response 88) (19) and TRIF [TIR (Toll IL-1R)-domain containing adaptor inducing Interferon-β (20)], along with the respective adaptor proteins MAL [MyD88-adaptor-like, also known as TIR-domain containing adaptor protein TIRAP (21)] and TRAM [TRIF-related adaptor molecule (22)]. TLR4 is able to induce different sets of pro-inflammatory responses: rapid MyD88-dependent induction of cytokines which starts at the plasma membrane, and TLR4/MD-2/LPS internalization-driven induction of type I interferons which depends on TRIF and originates from endosomal membranes. Although TLR4 has long been considered an important therapeutic target, very few structurally defined TLR4 - activating molecules have been developed so far. The reason for this deficiency resides in rather intricate and multifaceted TLR4-LPS structure-activity relationships which adds to the complexity in designing suitable TLR4 activating ligands.

**Figure 1:**
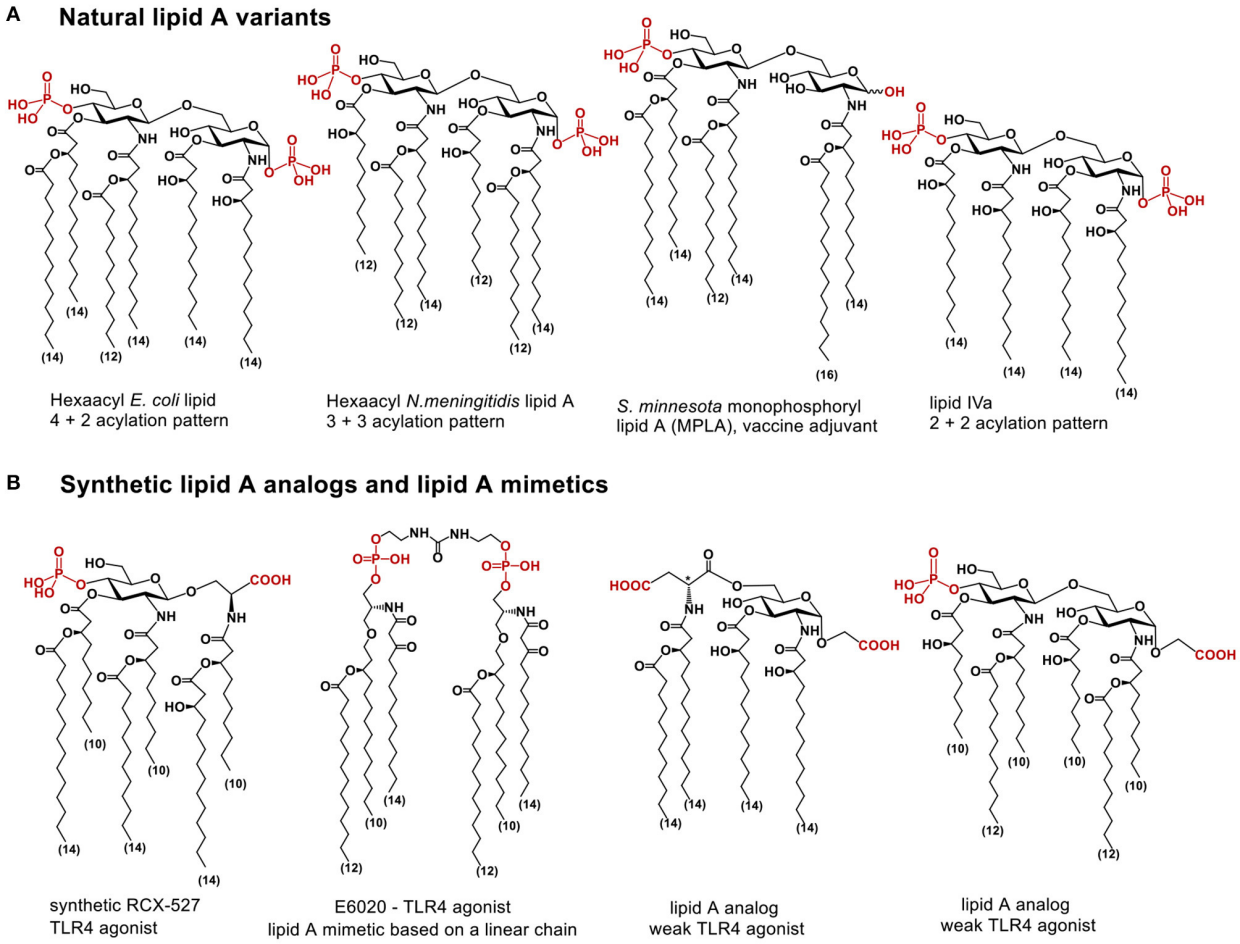
**(A)** chemical structure of lipid A; **(B)** lipid A analogs and lipid A mimetics based on flexible scaffolds (linear chain, or monosaccharide linked to an aglycon, or β(1 → 6)-linked diglucosamine).

The chemical structure of lipid A relies on a β(1 → 6) linked disaccharide backbone (βGlcN(1 → 6)GlcN) which is highly conserved in most bacterial species. The diglucosamine skeleton possesses one or two phosphate groups at positions 1 and/or 4′ and bears varying number long-chain (*R*)-3-hydroxyacyl- or (*R*)-3-acyloxyacyl residues which are symmetrically or asymmetrically allocated within the diglucosamine backbone at positions 2,3- and 2′,3′ (Figure 1A) (1). Hexaacylated lipid A of *E. coli* and *N. meningitidis* possessing two (*R*)-3-acyloxyacyl and two (*R*)-3-hydroxyacyl residues variably distributed within β(1 → 6) diglucosamine backbone (4+2 and 3+3 acylation pattern, respectively) exhibit the utmost TLR4-activating capacity, whereas tetra- or pentaacylated lipid A variants can act as antagonists at human (h)TLR4 [such as lipid IVa (23), or non-pathogenic lipid A from *R. sphaeroides* (24)].

**Figure 2:**
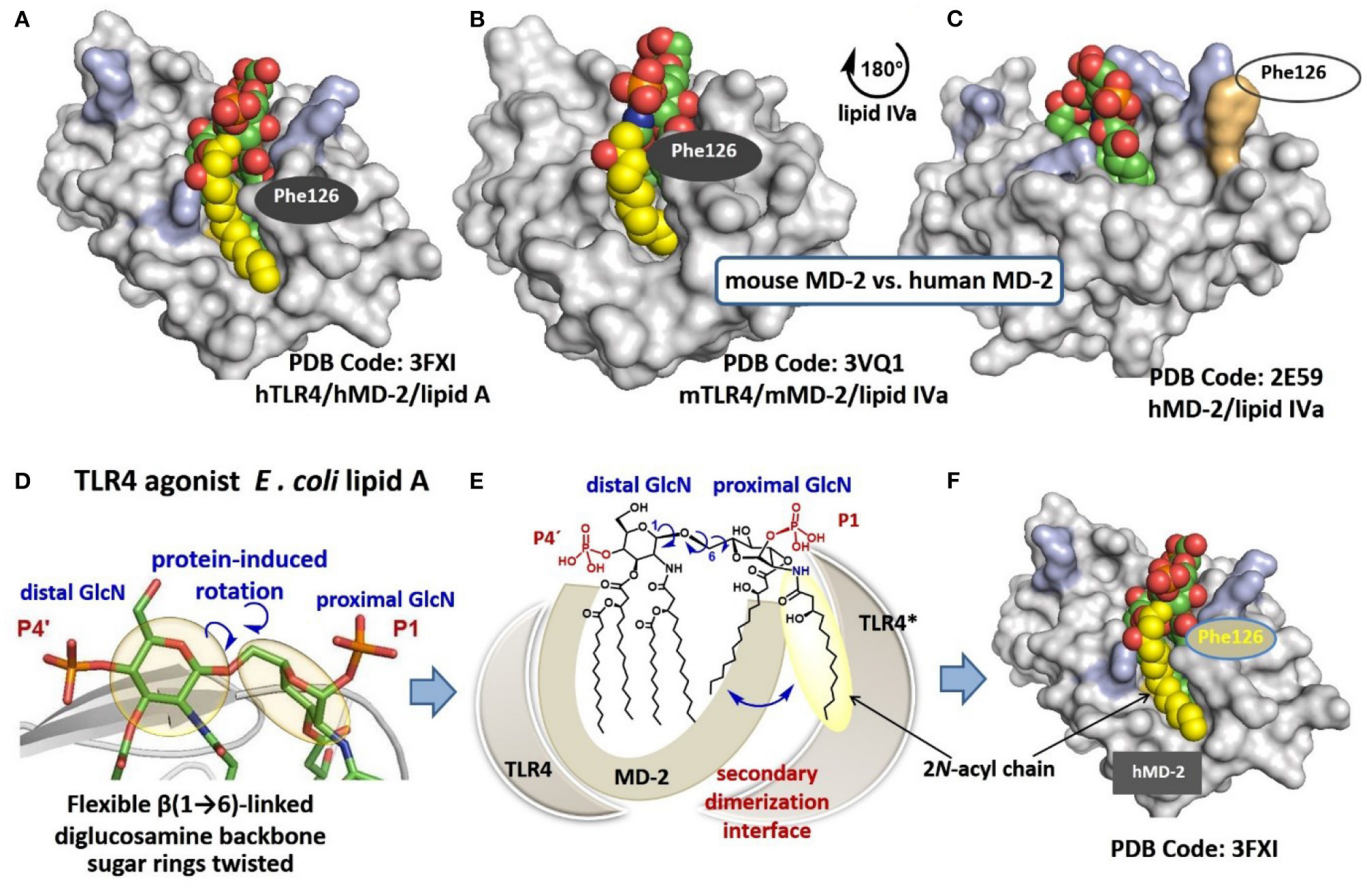
**(A)** crystal structure of hTLR4/MD-2-bound *E. coli Ra*-LPS (only lipid A portion is shown for clarity); **(B)** co-crystal structure of mTLR4/mMD-2-bound lipid IVa; **(C)** co-crystal structure of hTLR4/hMD-2-bound lipid IVa; **(D)** molecular shape of the β(1 → 6)-linked diglucosamine backbone of TLR4/MD-2 bound lipid A; **(E)** schematic representation of protein-induced adjustment of the molecular shape of the diglucosamine backbone of lipid A through rotation about β(1 → 6)-glycosidic linkage; **(F)** exposure of 2N-acyl chain of lipid A on the surface of MD-2 creates a hydrophobic patch for the interaction with the second receptor complex. Images were generated with PyMol and ChemDraw.

The co-crystal structures of hexaacylated *E. coli Re*- and *Ra*-LPS with mouse (m) or (h)MD-2/TLR4 complex, respectively, reveal that five long-chain acyl residues of lipid A (out of six) are incorporated into the hydrophobic Leu- and Phe-reach binding pocket of MD-2, while the sixth lipid chain is presented on the surface of the co-receptor protein MD-2 (Figure 2A) (14, 25). Binding of lipid A induces conformational rearrangement in MD-2 shifting the Phe126 loop inwards which, together with exposure of a lipid chain, creates a major hydrophobic interface for the dimerization with the second TLR4^*^/MD-2^*^/LPS complex (26, 27). Juxtapose, all lipid chains of hTLR4 antagonist ligands such as tetraacylated lipid IVa (23) (a biosynthetic precursor of *E. coli* lipid A) and synthetic drug-candidate Eritoran (28) are housed within the binding groove of hMD-2 (Figure 2C) and the Phe126 residue of MD-2 is exposed to solvent (23, 28) which prevents dimerization and abolishes the induction of pro-inflammatory signaling. Deduced from the co-crystal structures and molecular dynamic simulations, the Phe126 residue of MD-2 stabilizes presentation of the 6^th^ acyl chain of lipid A on the surface of the protein and serves as “hydrophobic switch” which either promotes (for agonists) or blocks (for antagonists) the dimerization and the formation of a hexameric receptor complex (14, 29). Ionic interactions between lipid A phosphate groups and positively charged side chains in MD-2 and TLR4^*^ (Lys, Arg) also substantially contribute to binding and dimerization (30). Remarkably, agonist lipid A variants are bound by MD-2 in inverted (by 180°) orientation compared to antagonist ligands. The position of the lipid A (±180°) within the binding cleft of MD-2 was demonstrated to be crucial for the expression of TLR4-mediated biological activity. Some lipid A variants exhibit species-specific TLR4-dependent activities which is largely determined by the binding orientation of lipid A in the binding groove of MD-2. For instance, tetraacylated lipid IVa acts as antagonist at hTLR4 but performs as an agonist at mTLR4 wherein it binds in an inverted by 180° orientation (similar to *E. coli* lipid A bound by hMD-2) and exposes one (out of four) lipid chain on the surface of mMD-2 (Figure 2B) (26, 31). The reason for exposure of a particular lipid chain (always 2*N*-acyl chain linked to the proximal GlcN moiety), as well as the basis for “inverted” positioning of the agonist lipid A in the binding pocket of MD-2 (rotation of the lipid A backbone by 180°) compared to antagonist lipid A variants is not yet understood.

Since the structure of the hydrophobic fatty chain region of lipid A belongs to primary determinants of “endotoxicity,” modulation of the length, number and distribution pattern of acyl chains was typically employed for alteration of cytokine-inducing capacity of genetically engineered (32, 33), isolated (34), and synthetic lipid A variants (35). Shortening or lengthening of only one acyl chain in the hydrophobic region of lipid A for 2xCH_2_ atoms frequently results in the accidental effects on TLR4-mediated signaling. This well-known dependency of TLR4 activation on the minimal aberrations in the length of lipid chains complicates prediction of biological activity of natural lipid A/LPS variants. Additionally, structural heterogeneity of lipid A isolates in respect to number of lipid residues and acylation pattern, along with potential contaminations by other bacterial components makes an application of lipid A/LPS isolated from bacterial cultures rather challenging for therapeutic use. As example, a pharmacological lipid A preparation—licensed vaccine adjuvant MPL® (clinical-grade MPL®, monophosphoryl lipid A manufactured by partial hydrolysis of *S. enterica serovar Minnesota Re*595 LPS)—represents a heterogeneous mixture of differently acylated counterparts exerting partial TLR4 antagonist activity which thwarts the adjuvant's efficacy (36). Besides, species—specific differences (human vs. mice) in lipid A recognition by the TLR4/MD-2 complex (26, 37) add to discrepancies in estimating therapeutic effect using experimental data obtained from *in vivo* models. To address these issues, we pursued a multidisciplinary approach combining synthetic glycochemistry, structural biology, and immunology.

Hydrophobic interaction between lipid chains are commonly dominant so that the six acyl chains of *E. coli* lipid A tend sticking together to form a solitary hydrophobic cluster. The exposure of a single 2N-acyl lipid chain on the surface of MD-2 must be, therefore, energetically unfavored unless there are some specific factors which assist in destabilization of hydrophobic interactions. We proposed that the exposure of 2N-acyl chain of *E. coli* lipid A on the surface of human or mouse MD-2 is enabled through intrinsic flexibility of a three-bond β(1 → 6) glycosidic linkage connecting two GlcN residues of the diglucosamine backbone of lipid A (Figure 2D). Protein driven rearrangement of the carbohydrate backbone of lipid A is facilitated by rotation about the β(1 → 6) linkage which results in a dramatic change in a 3D-molecular shape of the lipid A backbone seen in the co-crystal structures (14, 26). Accordingly, upon interaction with MD-2/TLR4, the proximal GlcN ring of lipid A (GlcN ring facing secondary dimerization interface) is relocated in a skewed orientation which might assists in weakening of intermolecular hydrophobic interactions between long-chain lipid residues (Figure 2E). This could allow a protrusion of 2N-acyl lipid chain (attached at the proximal sugar ring) on the surface of MD-2 without significant entropic loss (Figure 2F) (38). Thus, the inherently flexible β(1 → 6)-linked disaccharide backbone of lipid A readily adjusts its molecular shape upon interaction with the MD-2/TLR4 complex which is unveiled in the co-crystal structures. Taking into account intrinsic plasticity of MD-2 (39), the major conformational changes in both the ligand and the protein take place upon binding of the lipid A portion of LPS. This reduces the predictability of ligand-protein interaction such as the binding pose of a lipid A-like ligand (±180°), the deepness of insertion of lipid A (or a lipid A analog) into the binding groove of MD-2 (that significantly varies for agonist and antagonist lipid A), and the rearrangement behavior of Phe126 loop of MD-2 (29). Since the major lipid A recognizing protein MD-2 reveals multiple conformational states which exist in ligand-dependent equilibrium, the designing of TLR4 activating molecules using common structure-based approaches seems unsuitable for pharmacological manipulation of the TLR4 system.

**Figure 3:**
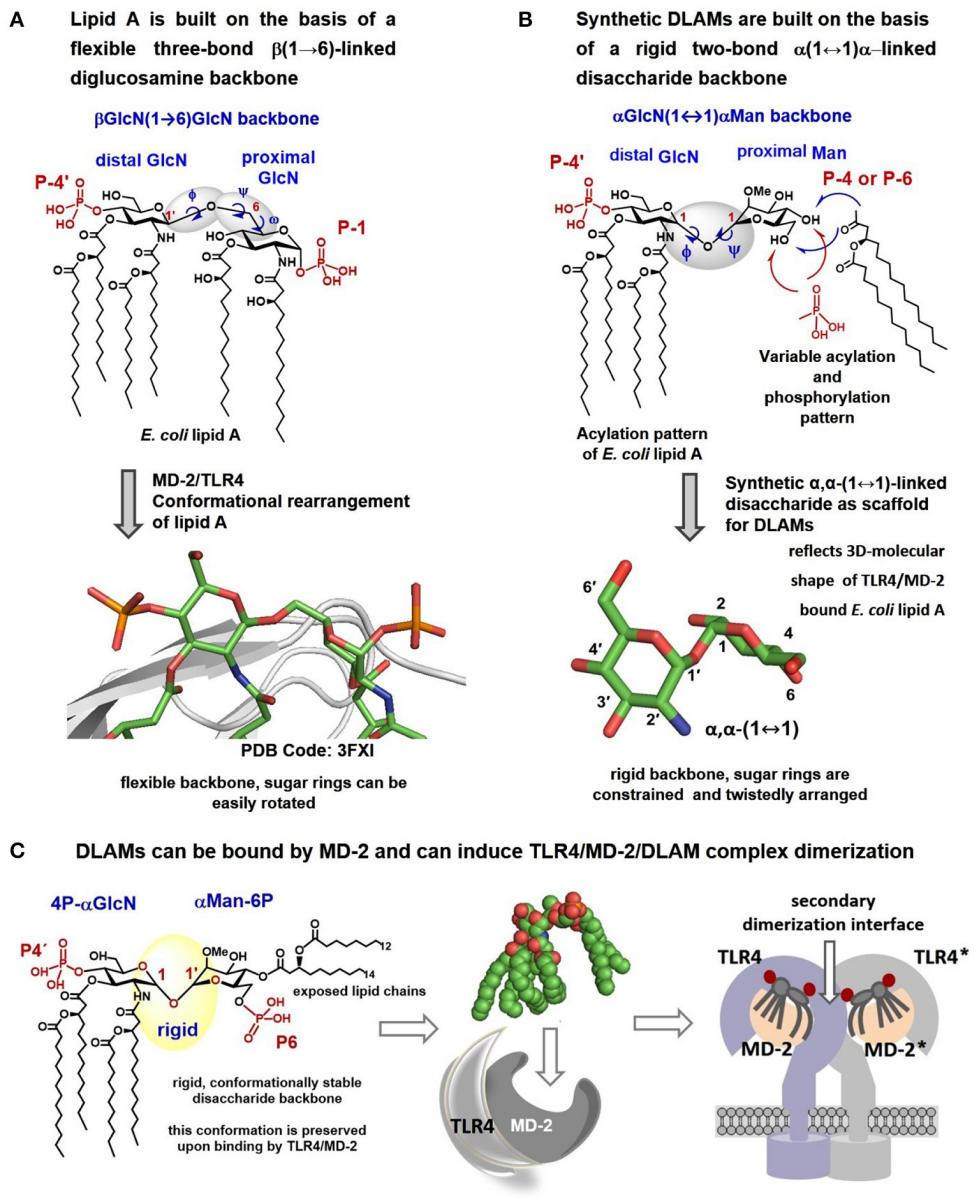
Crystal structure–based design of αα-GM-DLAMs. **(A)** Flexible three-bond β(1 → 6)-linked diglucosamine backbone of lipid A adopts twisted conformation upon binding by the MD-2/TLR4 complex (PDB code: 3FXI); **(B)** Flexible three-bond β(1 → 6)-linked diglucosamine backbone of lipid A was exchanged for a rigid two-bond α, α-(1↔1)-linked disaccharide scaffold to provide conformationally restricted disaccharide lipid A mimetics (DLAMs). The tertiary structure of the sugar backbone of αα-GM-DLAM reflects the molecular shape of the MD-2/TLR4 bound *E. coli* lipid A. **(C)** proposed mode of binding of DLAMs to the TLR4/MD-2 complex. Four acyl chains of distal GlcN ring (acylation pattern of *E. coli* lipid A) are supposed to be accommodated in the binding pocket of MD-2 and the proximal sugar ring is supposed to face the secondary dimerization interface. Images were generated with ChemDraw and PyMol.

We hypothesized that fixing the molecular shape of the diglucosamine backbone of lipid A in a skewed conformation found in the agonist TLR4/MD-2/lipid A co-crystal structures (PDB Code: 3FXI, 3VQ1, 3VQ2) using chemical methodologies could furnish lipid A mimetics that would be predictably and specifically bound by the TLR4/MD-2 complex in an “agonist” orientation (i.e., without ±180° uncertainty). Such lipid A mimetics should retain the major binding characteristics of *E. coli* lipid A, and simultaneously induce structural rearrangements in MD-2 which are required for inducing the receptor complex dimerization, i.e., moving the Phe126 loop inwards (Figure 3A) (26, 27, 40).

Using glycochemistry approaches we have synthetically assembled a non-reducing two-bond linked α,α-(1↔1′)-connected disaccharide to apply as surrogate for intrinsically flexible three-bond linked βGlcN(1 → 6)GlcN backbone of lipid A (Figure 3B) (41). High conformational rigidity of the α,α-(1↔1′)-linked disaccharides and their peculiar twisted molecular shape imitating the tertiary structure of the glucosamine backbone of TLR4/MD-2 bound *E. coli* lipid A (PDB Code: 3FXI, 3VQ2) were the key features our design relied on. The TLR4-mediated activity of disaccharide lipid A mimetics (DLAMs) relies, therefore, on both the 3D-tertiary structure of the non-reducing disaccharide backbone and on the number/length/distribution of lipid chains. Of note, all lipid A analogs and mimetics developed so far were based on either natural β(1 → 6)-linked diglucosamine backbone or even more flexible skeletons where one or both glucosamines were replaced by a linear aglycon (as in RCX 527 or E6020) (Figure 1B) (42). Also, a MD-2-specific TLR4 agonist having no similarity to LPS has been developed, although this peptidomimetic showed specificity to mouse TLR4 only (43).

The DLAMs are based on the conformationally restricted αGlcN(1↔1)αMan scaffold and therefore abbreviated α,α-GM-DLAMs (**α,α**-(1↔1)-linked **G**lucosamine-**M**annose-**D**isaccharide-**L**ipid **A**-**M**imetics). Here we validate the ability of synthetic TLR4 agonist α,α-GM-DLAMs to induce tailored activation of cellular pro-inflammatory responses in human and mouse TLR4/MD-2/CD14(±)-transfected cells, TPA-primed THP-1 cells, mouse macrophages (mBMDM), human mononuclear cells (MNC), and several types of lung epithelial cells. We demonstrate that DLAMs can induce robust TLR4-mediated release of cytokines in human and murine immune cells (MNC and BMDM, respectively) at picomolar concentrations, whereas the activation of TLR4-dependent responses in human airway epithelial cell lines and TPA-primed THP-1 macrophages required nanomolar DLAMs concentrations. We also assess the affinity of DLAMs to MD-2/TLR4 by competitive cellular assays using a known TLR4 antagonist which competes with DLAMs for the same binding site on MD-2. We demonstrate that graded and adjustable modulation of the pro-inflammatory signaling induced by DLAMs in human and murine cell lines can be achieved in a concentration-dependent manner through modification of the chemical structure of DLAMs.

Thus, disaccharide lipid A mimetics offer several advantages compared to natural lipid A variants in regard to (1) chemical stability: in DLAMs the inherently labile glycosidic phosphate group P-1 is replaced by a stable secondary phosphate group; (2) chemical purity: the highest purity (99.9%) and chemical homogeneity of DLAMs is confirmed by multiple analytical techniques such as ^1^H-, ^31^P, ^13^C-NMR (Bruker 600 MHz), HRMS and MALDI-TOF; (3) biological purity: DLAMs are produced by chemical synthesis which is performed starting from glucosamine by application of organic solvents and chemical reagents exclusively; all synthetic intermediates and target compounds are carefully isolated and thoroughly purified using chromatography in organic solvents which guaranties the absence of any biological contaminations. This renders the immunobiological data obtained with DLAMs to absolutely reliable, highly trustable and reproducible. Importantly, modulation of cellular pro-inflammatory responses induced by synthetic DLAMs has been independently studied and confirmed by four European laboratories.

## Methods

### Synthetic α,α-GM-DLAMs

**Figure 4:**
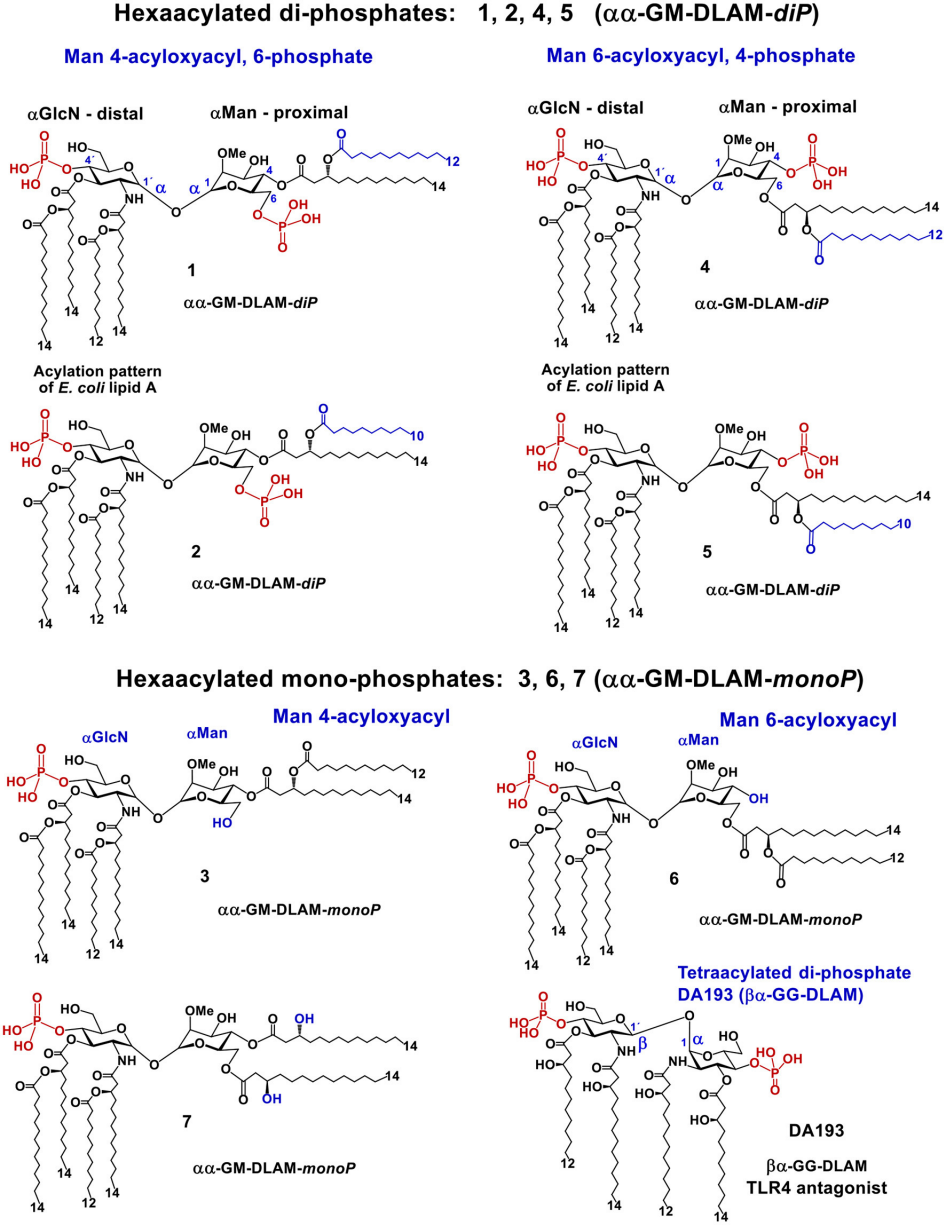
Chemical structure of lipid A mimetics based on the αGlcN(1↔1)αMan scaffold (αα-GM-DLAMs).

The acylation and phosphorylation pattern of the GlcN fragment of DLAMs (corresponding to distal GlcN ring of lipid A) reflects the one of *E. coli* lipid A (two acyloxyacyl residues at positions 2′ and 3′, and the phosphate group P-4′), whereas the second sugar (Man; mannose, which substitutes the proximal GlcN ring of lipid A) owns only two lipid chains (acyloxyacyl residue) and one/none phosphate groups that are variably linked at position 4 or 6 (Figure 4). To assess the relative contribution of hydrophobic and electrostatic interactions provided by acyl chains and phosphate groups, respectively, we synthesized four different groups of DLAMs: diphosphates having a second phosphate group at position 6 of the mannose moiety: αα-GM-DLAM-*di*P **1** and **2**; diphosphates entailing the second phosphate group at position 4 (Man): αα-GM-DLAM-*di*P **4** and **5**, whereas the length of secondary acyl chain attached at position 4 or 6, respectively, varied between C_12_ for DLAMs-*di*P **1** and **4** and C_10_ for DLAMs-*di*P **2** and **5**. The monophosphate DLAMs differed in the sites of attachment of the acyloxyacyl chain: DLAM-*monoP* **3**, a monophosphate counterpart of DLAMs-*di*P **1**, was lipidated at position 4, whereas DLAM-*monoP* **6**, a monophosphate counterpart of DLAMs-*di*P **4**, entailed the acyloxyacyl chain at position 6 (Figure 4).

Upon DLAMs interaction with the receptor complex, the four lipid chains at the distal GlcN ring are assumed to be captured and tightly bound by the hydrophobic binding pocket of MD-2, whereas the mannose ring bearing two lipid chains and one (or none) phosphate group is supposed to face the secondary dimerization interface (Figure 3C) with lipid chains either buried in the pocket or exposed on the surface of MD-2. Upon binding by the TLR4/MD-2 complex, the conformation of a highly rigid disaccharide backbone of DLAMs is preserved (i.e., cannot be affected by protein binding) in contrast to an easily adjustable inherently flexible β(1 → 6)-linked diglucosamine backbone of native lipid A (19). This was assumed to guarantee persistent hydrophobic and ionic interactions allowing for a tight binding of DLAMs to MD-2 in a pre-defined orientation which would results in ligand-driven protein rearrangement and formation of a stable dimerization interface. Thus, variations in the length of secondary acyl chains (e.g., C_14_ vs. C_12_) or alteration of phosphorylation/acylation pattern (exchange between positions 4 and 6) at Man moiety was hypothesized to alter the tightness and efficiency of TLR4 complex dimerization which could be then translated in deviating pro-inflammatory signaling.

### Solubilization of Synthetic Compounds, Preparation of Test Samples

Synthetically prepared DLAMs **1–7** were additionally purified by column chromatography on Bio-Beads SX-1 (Bio-Rad) prior to immunobiological evaluation. Synthetic procedures and all analytical data (^1^H-, ^31^P-, ^13^C- NMR, HRMS and MALDI-TOF data) of synthetic lipid A mimetics **1–3** (41) and DLAMs **4–7** (44) were reported earlier. Bio-Beads SX-1 resin is neutral, porous styrene divinylbenzene beads support (1% cross-linkage, 40–80 μm bead size, 600–14,000 MW exclusion range) for size exclusion chromatography of lipophilic biomolecules and biopolymers that require organic eluents. Solutions of DLAMs **1–7** (2–3 mg) in chloroform-toluene-methanol (3:2:1, v/v/v, 3 mL) were loaded onto a gel bed of Bio-Beads SX-1 (100 × 1 cm, pre-equilibrated in chloroform-toluene-methanol, 3:2:1, v/v/v) and eluted with the same solvent. Appropriate fractions were collected and concentrated to dryness under reduced pressure. The residues were redissolved in DMSO (2-3 mL, TLR4 grade, Sigma) and lyophilized. Compounds **1–7** (2.0 mg) were dissolved in DMSO (TLR4 grade, Sigma) to provide 1 mg/mL solutions which were subsequently aliquoted (0.1 mg, 100 μL of DLAM solution in DMSO) in 1 mL glass vials and lyophilized. The content of the vial (0.1 mg of compounds **1–7**) was reconstituted in DMSO (100 μL) under vortex (10 min). Aliquots (50 μL) were placed in 2–3 mL glass vials and diluted with 450 μL of cell medium (RPMI or DMEM) supplemented with 10% FCS (fetal calf serum) under vortex (10 min) to provide 500 μL of a 100 μg/mL stock solution (Stock I). Stock I was used for further dilutions with aqueous buffers or cell medium (as indicated below for each particular experiment) to provide aqueous solutions of compounds **1–7** which were used in the dose-dependent cell activation assays. Glass vials were used for all dilutions. The final concentration of DMSO in the cell cultures did not exceed 0.005% for DLAMs **1, 2, 4**, and **5** and 0.05% for **3, 6, 7**, and DA193. Stability and integrity of DLAMs **1–7** in aqueous buffers (100 μg/mL) at 37°C at 12, 24, and 36 h incubation time, as well as the stability in aqueous solution at 4°C during 1, 2, and 4 weeks of storage was confirmed using MALDI-TOF spectroscopy.

### Induction of Secreted Embryonic Alkaline Phosphatase (SEAP) in HEK-Blue (hTLR4/MD-2/CD14—Transfected HEK293) Cells

**Figure 5:**
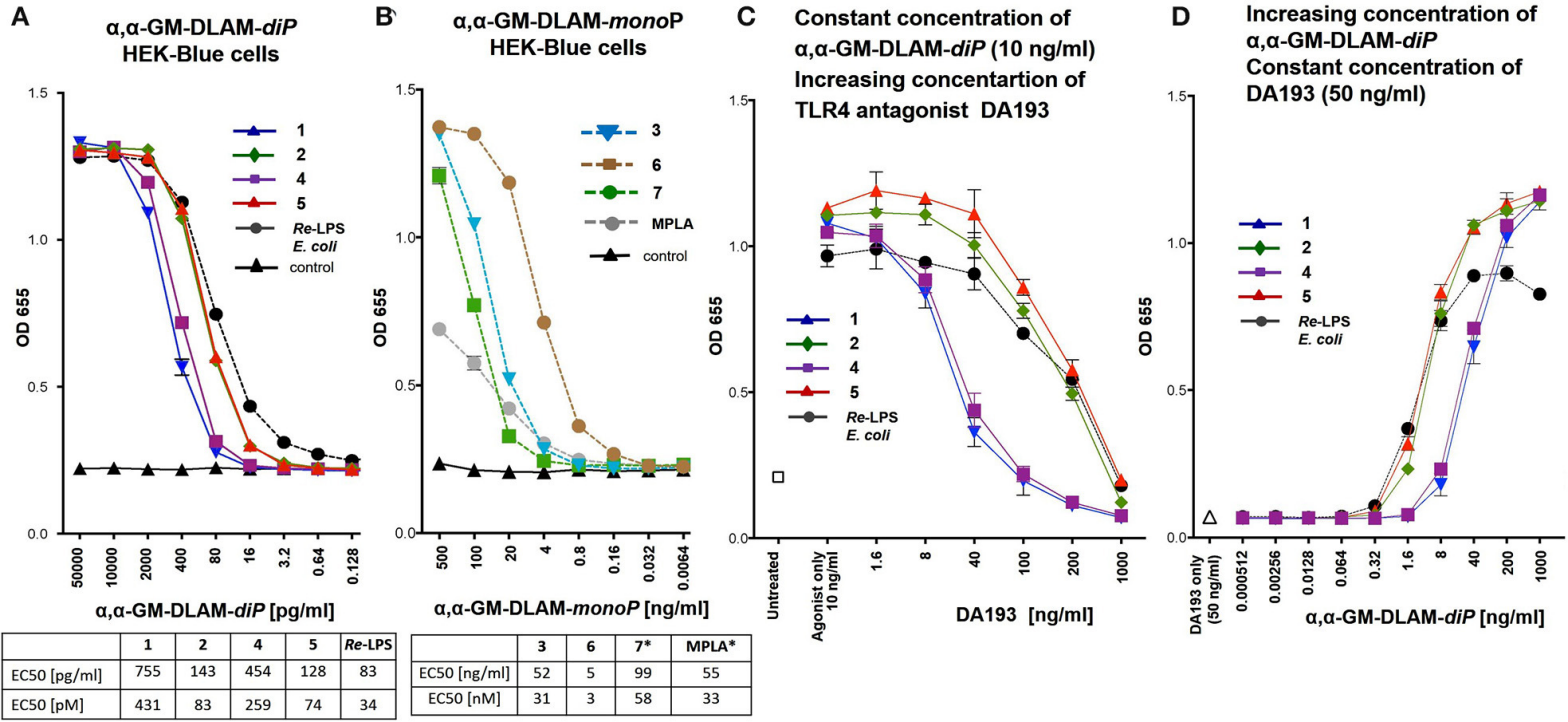
Dose-dependent activation of TLR4 signaling in hTLR4/hMD-2/hCD14 transfected HEK293 cells (HEK-Blue) induced by **(A)** αα-GM-DLAM-*diP* **1, 2, 4, 5** compared to *E. coli Re*-LPS; **(B)** αα-GM-DLAM-*monoP* **3, 6, 7** compared to *S. minnesota* MPLA; EC50 in pM were calculated based on following MW: DLAM 1 = 1751 Da, DLAM 2 = 1723 Da, DLAM 4 = 1751 Da, Fa148 = 1723 Da, *E. coli Re*-LPS = 2200 Da, DLAM 7 = 1715 Da, DLAM 3 = 1671 Da and DLAM 6 = 1671 Da; *EC50 calculation is subject to increased uncertainty due to limited data points at maximal response. **(C)** Dose-dependent inhibition of the DLAMs–induced TLR4 signaling (fix concentration of TLR4 agonist 10 ng/ml) by increasing concentration of synthetic TLR4 antagonist DA193 (competitive inhibition assay). **(D)** Dose-dependent inhibition of the DLAMs–induced TLR4 signaling (increasing concentration of TLR4 agonist DLAM) by fix concentration of synthetic TLR4 antagonist DA193 (50 ng/ml) (competitive inhibition assay). Data were combined from *n* = 3 independent experiments, error bars indicate standard error of the mean.

HEK293 stably expressing human TLR4, MD-2, CD14, and a secreted NF-κB dependent reporter were purchased from InvivoGen (HEK-Blue hTLR4). Growth conditions and endotoxicity assay were set as recommended by InvivoGen. *E. coli* serotype R515 *Re*-LPS and *S. minnesota* R595 MPLA were purchased from InvivoGen. Stock solutions of DLAMs were prepared as indicated above, further dilutions were made with DMEM supplemented with 10% FCS. The cells were stimulated with DLAMs **1-7** or MPLA/Re-LPS at the indicated concentrations (Figures 5A,B). The compounds were added in a total volume of 20 μl to 25000 HEK-Blue hTLR4 cells in 180 μl per well of a 96-well-plate. In the competition experiment [Figure 5C: fix concentration of DLAMs (10 ng/ml), increasing concentration of TLR4 antagonist DA193 (45)] the DLAMs were added in a total volume of 10 μl at 10 ng/ml together with the indicated concentration of DA193 (always in 10 μl), 25000 HEKBlue hTLR4 cells in 180 μl were added and the plate was incubated for 20–24 h at 37°C and 5% CO_2_. In the competition experiment (Figure 5D: fix concentration of TLR4 antagonist DA193 (50 ng/ml), increasing concentration of DLAMs), 25000 HEK-Blue hTLR4 cells in 180 μl per well of a 96-well-plate were preincubated with DA193 (50 ng/ml in a total volume of 10 μl) and stimulated with DLAMs which were added in a total volume of 10 μl at the indicated concentration, and the plate was incubated for 20 h at 37°C and 5% CO_2_. Detection was performed using standard QUANTI-Blue protocol (InvivoGen) as follows: challenged cells supernatant (20 μl) was incubated with a detection reagent (180 μl, QUANTI-Blue, InvivoGen). Plates were incubated at 37°C and 5% CO_2_ and color developed was measured at 650 nm using a spectrophotometer (SpectraMAX 190). Data were combined from *n* = 3 independent experiments, error bars indicate standard error of the mean. The EC50 values were calculated in [pg/ml] and [pM] units, the standard deviation of EC50 values are <10% of the average.

### Stimulation of Transient Human TLR4/MD-2/CD14^(±)^ Transfected HEK293 Cells With αα-GM-DLAMs

**Figure 6:**
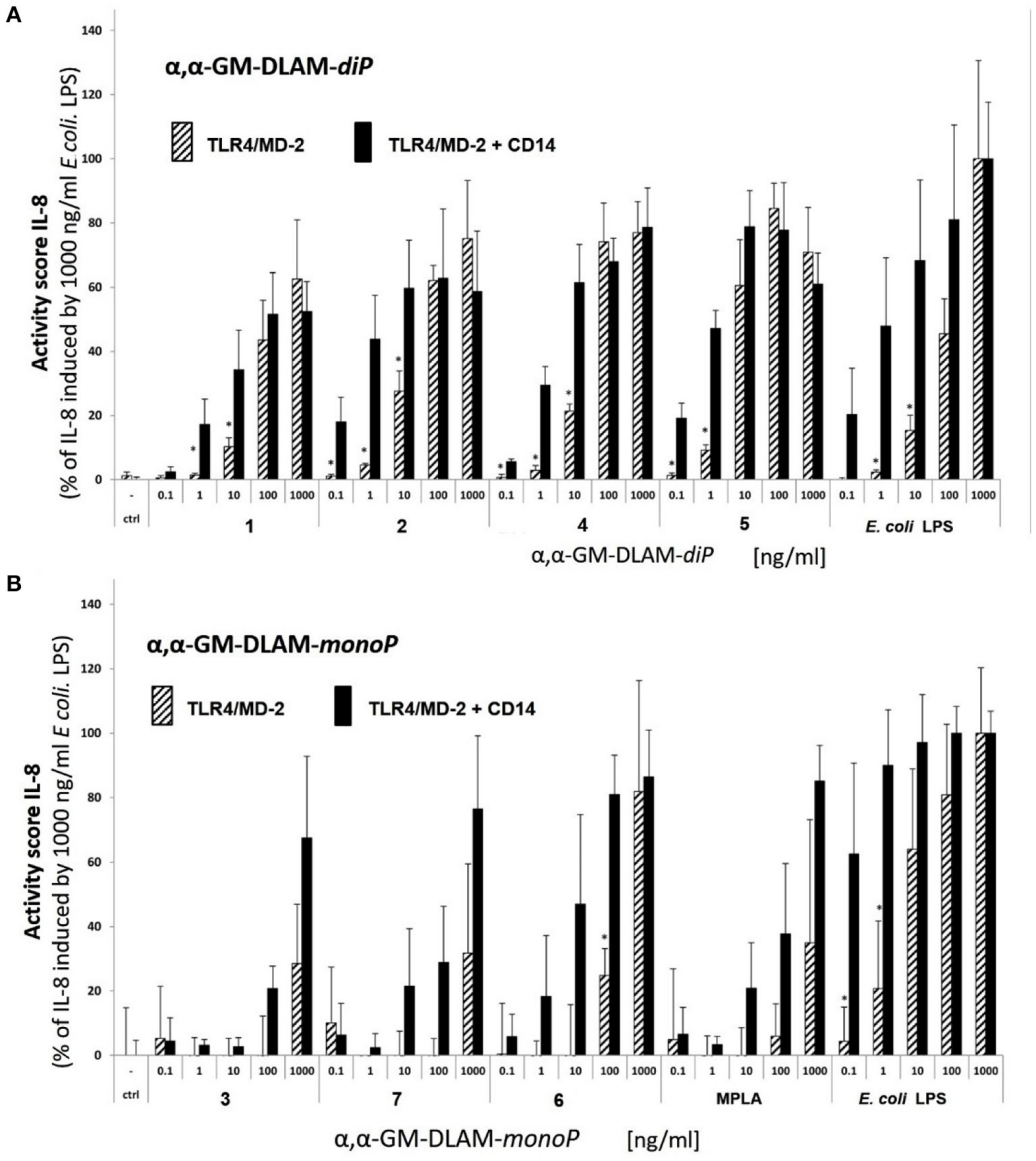
Dose-dependent activation of TLR4 signaling induced by **(A)** αα-GM-DLAM-*diP* in TLR4/MD-2/CD14^**(±)**^—transfected HEK293 cells comparted to *E. coli* LPS; **(B)** αα-GM-DLAM-*monoP* in TLR4/MD-2- or TLR4/MD-2/CD14—transfected HEK293 cells comparted to *E. coli* LPS and MPLA. Significantly different results (CD14(–) compared to CD14(+) cells for each concentration and compound individually) are indicated; *p < 0.5. Data were combined from *n* = 2 independent experiments, error bars indicate standard deviation of the mean. Calculation of statistical significance (GraphPad Prism 8) was determined using multiple *t*-tests (comparing each compound in CD14 (–) vs. CD14 (+) cells) and the Holm-Sidak method, with alpha = 0.05.

HEK293 cells were transfected for 24 h with plasmids coding for human TLR4 (kind gift of P. Nelson, USA), human MD-2 (kind gift of K. Miyake, Tokyo, Japan), and human CD14 (kind gift of D. Golenbock, Worcester, USA) using Lipofectamin2000 (Invitrogen GmbH, Karlsruhe, Germany) according to the manufacture's instruction. Solutions of DLAMs **1–7** were prepared as described above using DMEM cell medium supplemented with 10% FCS. Next, transiently transfected cells were stimulated with increasing concentrations of compounds **1–7** or *E. coli* O111:B4 LPS (a kind gift of Otto Holst, Research Center Borstel) and *S. minnesota* R595 MPLA (Invivogen) as positive controls for 20 h (Figure 6). Recombinant human TNF-α (kind gift of D. Männel, Regensburg, Germany) served as transfection-independent control. IL-8 production was measured by human IL-8 CytoSet ELISA (Invitrogen) according to the manufacture's instruction. Data were combined from *n* = 2 independent experiments, error bars indicate standard deviation of the mean. GraphPad Prism 8 was used for the calculation of statistical significance in Figure 6, which was determined using multiple *t*-tests (comparing each compound in CD14 (–) vs. CD14 (+) cells) and the Holm-Sidak method, with alpha = 0.05, for computing statistical significance. Each row was analyzed individually, without assuming a consistent SD.

### Stimulation of Transient Mouse TLR4/MD-2 Transfected HEK293 Cells (Dual Luciferase Reporter Assay)

**Figure 7:**
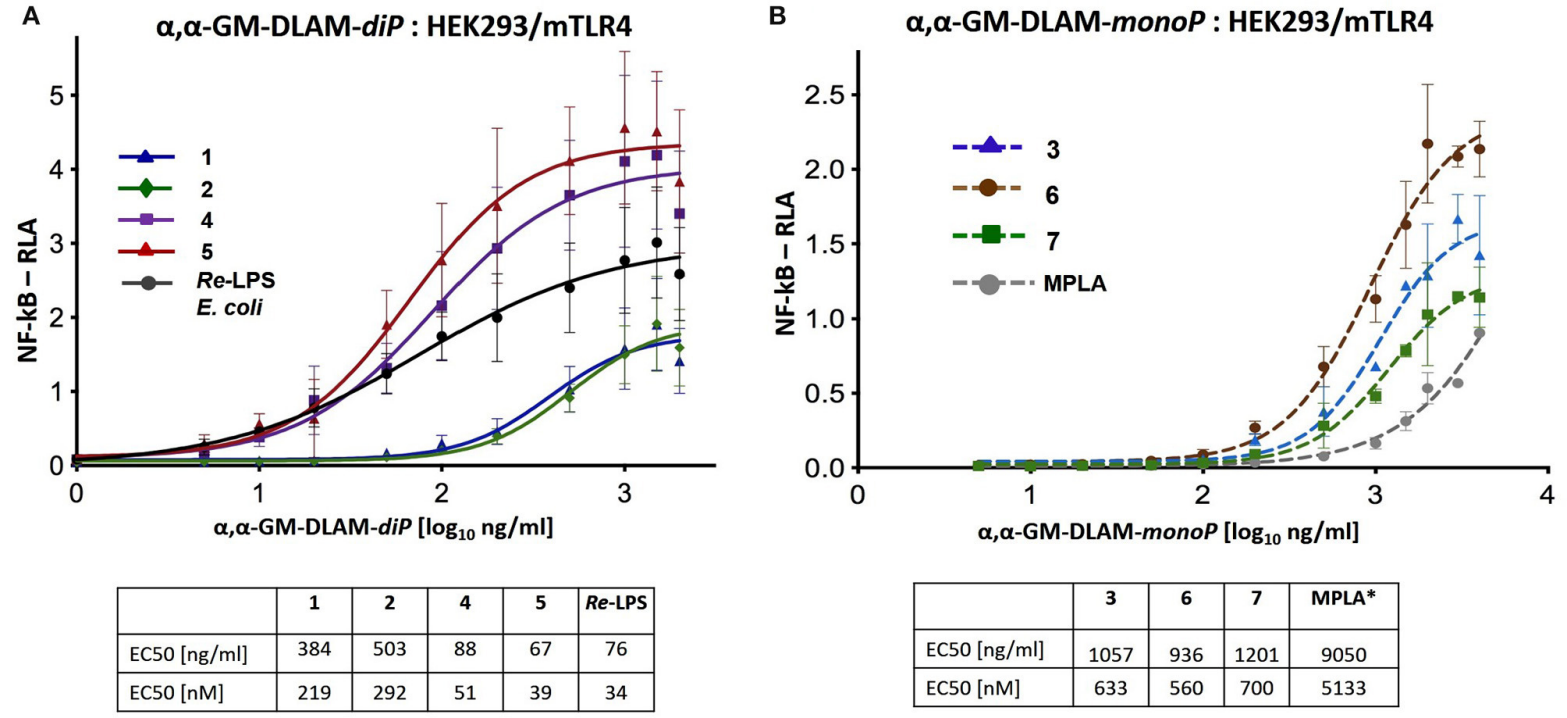
Dose-dependent activation of TLR4 signaling in mTLR4/mMD-2 transfected HEK293 cells induced by **(A)** αα-GM-DLAM-*diP* **1, 2, 4, 5** compared to *E. coli Re*-LPS; **(B)** αα-GM-DLAM-*monoP* **3, 6, 7** compared to *S. minnesota* MPLA. Non-linear curve fit (variable slope, four parameters) was calculated using GraphPad Prism. EC50 in [nM] were calculated based on the following MW: DLAM 1 = 1751 Da, DLAM 2 = 1723 Da, DLAM 4 = 1751 Da, Fa148 = 1723 Da, *E. coli Re*-LPS = 2200 Da, DLAM 7 = 1715 Da, DLAM 3 = 1671 Da and DLAM 6 = 1671 Da; *EC50 calculation is subject to increased uncertainty due to limited data points at maximal response. Data were combined from *n* = 3 independent experiments; error bars indicate standard error of the mean. GraphPad Prism 9 was used to perform statistical analysis.

HEK293 cells (human embryonic kidney 293 cells) were kindly provided by Dr. J. Chow (Eisai Research Institute, Andover, MA, USA). Expression plasmid containing the mouse MD-2 was a gift from Dr. Y. Nagai (University of Tokyo, Japan). Expression plasmid for mouse TLR4 sequence was purchased from InvivoGen and the Renilla luciferase phRL-TK plasmid was purchased from Promega (USA). The MD-2 encoding nucleotide sequences were cloned into pEF-BOS vector with Flag and His tags on the C-terminal. The nucleotide sequences encoding TLR4 were cloned into pUNO vector with C-terminal HA tag. Transfection reagent JetPEI was purchased from Polyplus-Transfection (Illkirch, France) and was used according to the manufacturer's instructions. HEK293 cells were seeded in 96-well-plates (Costar plates, Corning, NY, USA) in DMEM supplemented with 10% FBS at 2,5 × 10^4^ cells/well. Plates were incubated overnight at 37°C in a humidified atmosphere (5% CO_2_). The next day, cells were co-transfected with pEF-BOS-MD-2 (10 ng), pUNO-TLR4 (1 ng), NF-κB-dependent luciferase (75 ng) and constitutive Renilla (15 ng) reporter plasmids using JetPEI transfection reagent (7,5 molar polyethylenimine, pH 7,5). Cells were stimulated 6 h after transfection with DLAMs, *Re*-LPS (Invivogen) or MPLA (Invivogen) that were extensively vortexed immediately prior to stimulation and added as 10 μL solutions to reach final concentrations indicated in Figure 7. DMEM/10% FBS with 0,01% DMSO was used for mock stimulation (negative control). Total volume of the well after stimulation was 130 μL. All cells were lysed after 16 h of stimulation in 1x reporter assay lysis buffer (Promega, USA) and analyzed for reporter gene activity using a dual-luciferase reporter assay system. The NF-κB-dependent luciferase activity of each sample was normalized for constitutive Renilla activity for the calculation of RLA (Relative luciferase activity). Data were combined from *n* = 3 independent experiments; error bars indicate standard error of the mean. GraphPad Prism 9 was used to perform statistical analysis. Concentration values (ng/ml) were transformed into Log(X) to be included in the linear regression model (log(x) vs. response; variable slope; least squares fit). The EC50 values were calculated in [ng/ml] and [nM] units.

### Activation of Human Mononuclear Cells (MNC) by αα-GM-DLAMs

**Figure 8:**
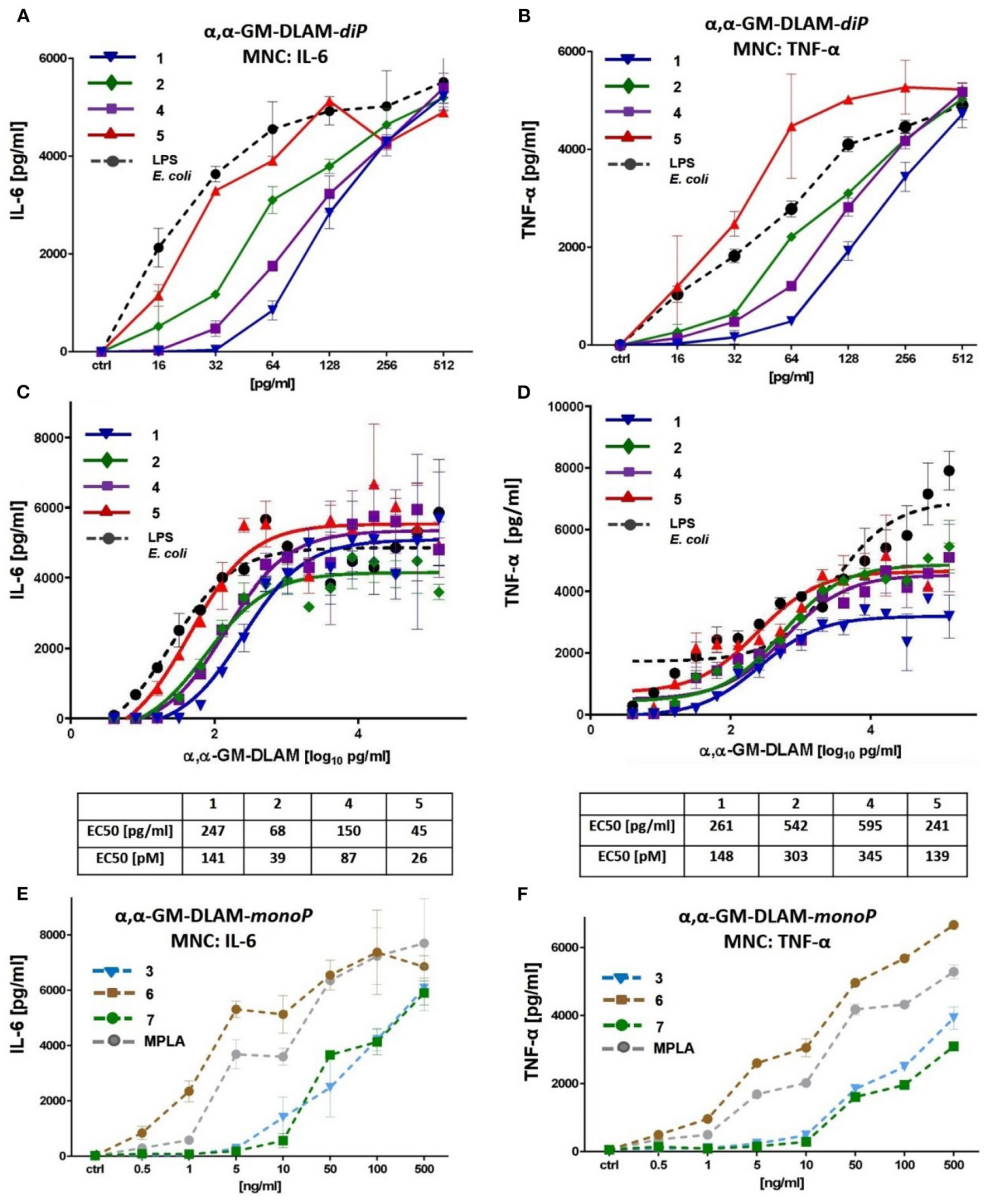
Dose-dependent induction of IL-6 and TNF-α induced by DLAMs in MNC. **(A)** induction of IL-6 by low concentrations (0.5–500 ng/ml) of αα-GM-DLAM-*diP*; **(B)** induction of TNF-α by low concentrations (0.5–500 ng/ml) of αα-GM-DLAM-*diP*; **(C)** dose-dependent expression of IL-6 by high concentrations (1–5000 ng/ml) of αα-GM-DLAM-*diP*; **(D)** dose-dependent expression of TNF-α by high concentrations (1–5000 ng/ml) of αα-GM-DLAM-*diP*; EC50 in [pM] were calculated based on the following MW: DLAM 1 = 1751 Da, DLAM 2 = 1723 Da, DLAM 4 = 1751 Da, Fa148 = 1723 Da, DLAM 7 = 1715 Da, DLAM 3 = 1671 Da and DLAM 6 = 1671 Da. **(E)** induction of IL-6 by low concentrations (0.5–500 ng/ml) of αα-GM-DLAM-*monoP* compared to *S. minnesota* MPLA; **(B)** induction of TNF-α by low concentrations (0.5–500 ng/ml) of αα-GM-DLAM-*monoP* compared to *S. minnesota* MPLA. Data shown are exemplary for *n* = 3 independent experiments, error bars indicate standard deviation of the mean.

MNC (peripheral human blood mononuclear cells) were prepared from heparinized blood from healthy volunteers by gradient centrifugation (Biocoll, Merck) and were subsequently incubated in 96-well-tissue culture plates at a volume of 150 μL and a concentration of 1 × 10^6^/mL using as medium RPMI-1640 supplemented with 100 U/mL penicillin (PAA Laboratories GmbH), 100 μg/mL streptomycin (PAA Laboratories GmbH), and 10% FCS (Merck Millipore, Biochrom AG, Germany). Solutions of DLAMs **1–7** were prepared from stock solutions in DMSO as described above using PRMI cell medium supplemented with 10% FCS. Cells were then stimulated with increasing concentrations of compounds **1–7** or *E. coli* O111:B4 LPS (Invitrogen) as positive control for αα-GM-DLAM-*diP* **1, 2, 4, 5**, and MPLA as positive control for αα-GM-DLAM-*monoP* **3, 6, 7** (Figure 8). After a culture period of 20 h at 37°C, culture supernatants were harvested and the IL-6 and TNF-α content was determined using an ELISA according to the manufacturers' protocol (Invitrogen). Data shown are exemplary for *n* = 3 independent experiments, error bars indicate standard deviation of the mean. For EC50 values, GraphPads Prism8 non-linear fit model [log[agonist] vs. response (three parameters)] with least square regression has been used. EC50 values are given in [pg/ml] as well as in [pM], the standard deviation of EC50 values are <10% of the average.

**Figure 9:**
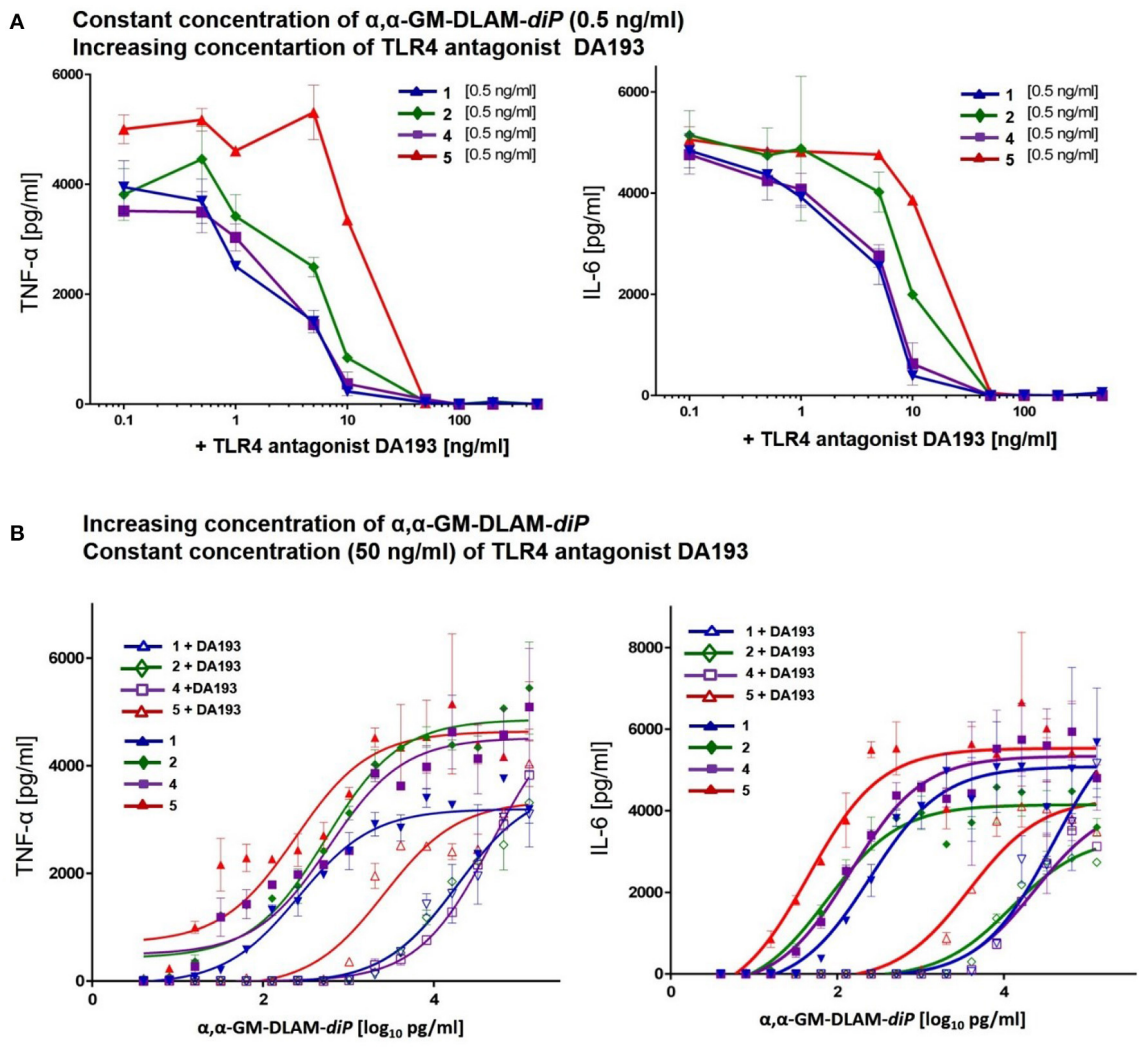
Competitive dose-dependent inhibition of pro-inflammatory signaling induced by αα-GM-DLAM-*diP* **1, 2, 4, 5** in MNC. Residual cytokine release (TNF-α, IL-6) monitored after: **(A)** MNC were pretreated with increasing concentrations of TLR4 antagonist DA193 and stimulated with 0.5 ng/ml of DLAMs **1, 2, 4, 5**; **(B)** MNC were pretreated with 50 ng/ml TLR4 antagonist DA193 and stimulated with increasing concentrations of DLAMs **1, 2, 4, 5**. Data shown are exemplary for *n* = 3 independent experiments, error bars indicate standard deviation of the mean.

In the competition experiments with simultaneous application of the TLR4 antagonist DA193 (45) cells were pre-incubated with increasing concentrations (0.1–100 ng/ml) of synthetic TLR4 antagonist DA193 for 15 min and subsequently stimulated with a constant concentration of DLAMs **1, 2, 4**, or **5** (0.5 ng/mL, Figure 9A). Alternatively, cells were pre-incubated with synthetic TLR4 antagonist DA193 (50 ng/mL) for 15 min. and subsequently stimulated with increasing concentration of DLAMs **1, 2, 4**, or **5** (1–1000 pg/mL) for 20 h. at 37°C. The culture supernatants were harvested, and the cytokine content (IL-6 and TNF-α) was determined using ELISA (Invitrogen) according to the manufacturers' instructions. Data shown are exemplary for *n* = 3 independent experiments, error bars indicate standard deviation of the mean.

### Stimulation of TPA-Primed THP-1 Cells by αα-GM-DLAMs

**Figure 10:**
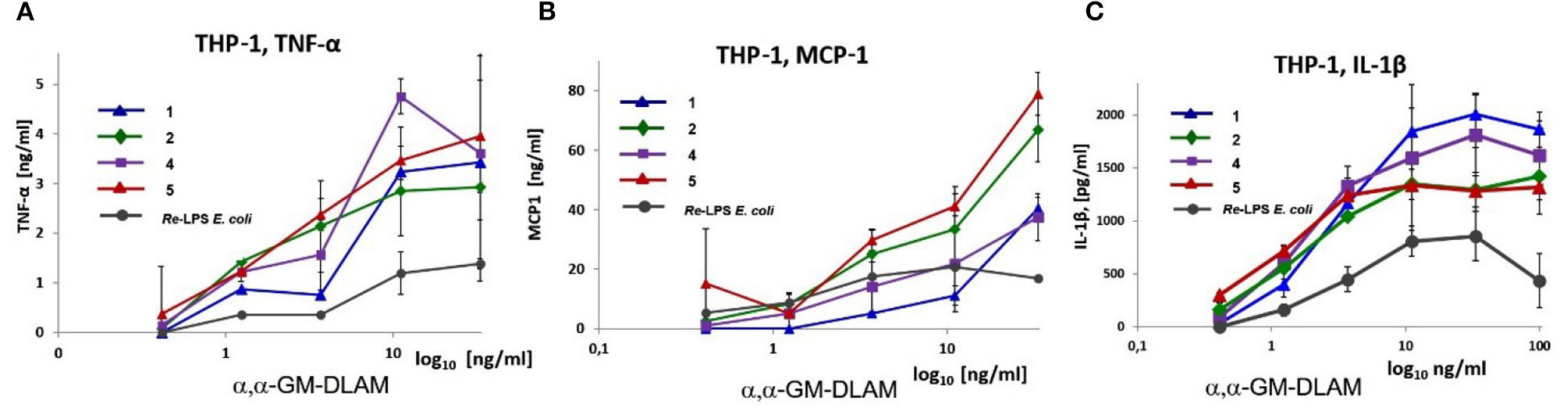
Dose-dependent expression of pro-inflammatory cytokines: **(A)** TNF-α, **(B)** MCP-1 and **(C)** IL-1β induced by αα-GM-DLAM-*diP* in TPA primed THP-1 cell line compared to *E. coli Re*-LPS. Data are the mean of *n* = 2 samples and are representative for *n* = 2 independent experiments. Error bars indicate standard deviation of the mean.

The THP-1 cells (ATCC) were grown in cell-culture medium RPMI-1640 (Life Technologies) supplemented with 100 U/mL penicillin, 2 mM L-glutamine, 100 μg/mL streptomycin, and 10% FCS. Cells were seeded in a 96 well-plate at 10^5^ cells/well in 150 μl complete medium and stimulated by treatment with 200 nM TPA (12-O-tetradecanoylphorbol-13-acetate, Sigma) for 24 h to induce the differentiation into macrophage-like cells (46). On the next day the cells were washed twice with complete culture medium to discard the cells that did not adhere, refreshed with 200 μl complete medium and left for 1 h to recover. Solutions of DLAMs **1–7** were prepared starting from 1 mg/mL stock solutions in DMSO as described above using RPMI-1640-cell-culture medium supplemented with 10% FCS. Cells were stimulated with αα-GM-DLAM-*diP* **1, 2, 4**, and **5** at the indicated concentration or with *E. coli Re*-LPS (InvivoGen), which were added as solutions in 10 μl complete medium (Figure 10). After stimulation the final volume of the well-reached 220 μl. The cells were incubated for 18 h and the supernatants were analyzed for IL-1β, TNF-α, and MCP-1 by ELISA (BD Biosciences). Data are the mean of *n* = 2 samples and are representative for *n* = 2 independent experiments. Error bars indicate standard deviation.

### Activation of Human Bronchial Epithelial Cells by αα-GM-DLAMs

Human lung epithelial cell line Calu-3 (ATCC) or human brochoepithelial cell line BEAS-2B (ATCC) were seeded in 96-well-plates at 10^5^ cells/well in 100 μl of complete medium (RPMI1640 (PAA), 1% PS (PAA), 10% FCS (Biochrom, Berlin, Germany). On the next day, cells were washed once with complete medium and stimulated with increasing concentrations of DLAMs **1–7** or *E. coli Re-*LPS as positive control, such that after stimulation the total volume of the well-reached 200 μl. The cells were incubated for 20 h and the supernatants were analyzed for cytokines (IL-8 and IL-6) by ELISA (Invitrogen). Data were combined from *n* = 2 independent experiments, error bars indicate standard error of the mean.

### Expression of cytokines in Bone Marrow-Derived wt Mouse Macrophages (BMDM) Stimulated by αα-GM-DLAMs

BMDM (bone-marrow-derived microphages) were obtained from the bone marrow of C57BL/6 OlaHsd mice. RPMI media was used to flash the bone marrow from femur and fibia. The erythrocytes were lysed for 15 min at 37°C with 0.88% ammonium chloride. Cell culture flasks were used to seed a single-cell suspension of the bone marrow cells (at a concentration of 1 × 10^6^ cells/mL); RPMI supplemented with 40 ng/mL recombinant MCSF and 20% FBS was used as cell medium. The cell culture medium was exchanged after 3 days. After 5 days the differentiated cells were trypsinized and counted. Next, the differentiated cells were seeded in 96-well-plates in RPMI supplemented with 10% FBS at a concentration of 3,5 × 10^4^ cells/well and left for 24 h. Afterwards, the cells were treated with DLAMs **1-7** solubilized as indicated above using broad concentration range of TLR4 agonists, *E. coli Re*-LPS (Invivogen), synthetic *E. coli* lipid A (Peptanova) or MPLA (Invivogen) for 16 h (**Figure 12**). The total volume of the well after stimulation was 120 μL. After 16 h incubation time, the supernatants were analyzed for mTNF-α and mIL-6 by ELISA (Ready-Set-Go ELISA kits, eBioScience). Data were combined from *n* = 3 independent experiments; error bars indicate standard error of the mean. GraphPad Prism 9 was used to perform statistical analysis. Concentration values [ng/ml] were transformed into Log(X) to be included in the linear regression model (log(x) vs. response; variable slope; least squares fit). The EC50 values are given in [pg/ml] and [pM] units.

### Expression of TNF-α in Bone Marrow-Derived TRIF-KO-, MyD88-KO-, and MyD88/TRIF-KO Mouse Macrophages (BMDM) Stimulated by αα-GM-DLAMs

Immortalized C57BL/6 wt and indicated knockout mouse macrophage cell lines were kindly provided by D.T. Golenbock (22) (Worcester, MA, USA) and propagated in RPMI medium (PAA, Linz, Austria) containing 10% FCS, 20 mM HEPES buffer, 2 mM L-Glutamin (both PAA, Linz, Austria) and 20 μg/ml gentamicin (Sigma, Deisenhofen, Germany). Subsequently, cells were stimulated with increasing concentrations of DLAMs **1-7** or *E. coli* O111:B4 LPS for 20 h (**Figure 13**). TNF-α production was measured by mouse TNF-α CytoSet ELISA (Invitrogen GmbH, Karlsruhe, Germany) according to the manufacture's instruction. Data shown are exemplary for *n* = 2 independent experiments, error bars indicate standard deviation of the mean.

## Results

### TLR4-Mediated Activation of NF-κB Regulated Signal Transduction Pathway by αα-GM-DLAMs

The capability of DLAMs to induce pro-inflammatory signaling in a TLR4-dependent manner was initially examined in hTLR4/MD-2/CD14 transfected human embryonic kidney 293 cells (HEK-Blue) by measuring the induction of secreted embryonic alkaline phosphatase (SEAP). The analysis of TLR4-mediated activation of NF-κB regulated signal transduction pathway by DLAMs was performed over a broad concentration range (0–500 ng/ml) and compared to responses induced by *E. coli Re*-LPS (Kdo_2_-lipid A) for DLAMs diphosphates **1, 2, 4**, and **5** and by MPLA for DLAMs mono-phosphates **3, 6**, and **7**. Generally, di-phosphate DLAMs **1, 2, 4**, and **5** (α,α-GM-DLAM-*diP*) exhibited TLR4-stimulatory activities in picomolar concentration range (Figure 5A), whereas the monophosphates **3, 6**, and **7** (α,α-GM-DLAM-*monoP*) were less potent and showed TLR4-mediated activities at nanomolar concentrations (Figure 5B). DLAMs **1** and **4** having 2 × CH_2_ longer acyl side chain were less efficient activators of NF-κB compared to DLAMs **2** and **5**. Thus, shortening secondary acyl chain facing secondary dimerization interface by 2 × CH_2_ resulted in enhanced TLR4-mediated signaling, whereas the sites of attachment of acyl chains and phosphate group (position 4 or 6 at Man moiety) did not exert significant effects on the efficiency of SEAP induction. Among monophosphates, 6-acyloxyacyl α,α-GM-DLAM-*monoP* **6** (lipid chain in position 6 of mannose, i.e., the lipid chain presumably presented at the secondary dimerization interface) induced significantly higher SEAP levels in HEK-Blue cells compared to the 4-acyloxyacyl DLAM **3** (lipid chain in position 4 of mannose). Notably, DLAMs-*monoP* were more efficient in inducing TLR4-dependent NF-κB signaling compared to monophosphoryl lipid A (MPLA, a licensed vaccine adjuvant).

To investigate the affinity of differently acylated bis-phosphorylated DLAMs to hMD-2/TLR4, a competitive inhibition assay using synthetic TLR4 antagonist DA193 (38, 45, 47) was performed (Figures 5C,D). HEK-Blue cells were incubated with α,α-GM-DLAM-*diP* **1, 2, 4**, or **5** (10 ng/ml—a concentration which induces the maximum level of NF-κB activation for all ligands) in the presence of increasing concentrations of DA193 (1–1000 ng/ml) and the residual SEAP levels were measured (Figure 5C). DA193 is a potent TLR4 antagonist based on a planar-arranged β,α-1,1′-linked diglucosamine scaffold which can compete with LPS for the binding site on MD-2 and can also displace LPS from the binding pocket (38, 45). The induction of SEAP stimulated by DLAMs **1** and **4** could be readily inhibited with DA193 (100 ng/ml), whereas the inhibition of NF-κB activation by DLAMs **2** and **5** required 10-fold higher concentration of DA193, which indicates superior binding affinity of DLAMs **2** and **5** to the hMD-2/TLR4 complex. The inverted experimental setting, when the cells were pre-incubated with a fix amount of TLR4 antagonist DA193 (50 ng/ml) and increasing concentrations of DLAMs were applied, demonstrated similar dependencies (Figure 5D).

Next, we studied the dependency of DLAMs-induced TLR4-mediated signaling on membrane CD14 (memCD14) by measuring IL-8 release in hTLR4/MD-2/CD14(±) transfected HEK293 cells. Co-transfection with memCD14 substantially enhanced the release of IL-8 by DLAM-*diP* (Figure 6A, solid black bars) through increasing the sensitivity of the TLR4/MD-2 complex to DLAMs by transferring the monomeric DLAM to MD-2 (48, 49). In the absence of memCD14 (Figure 6A, stripped bars) the expression of IL-8 dropped dramatically at lower doses of TLR4 agonists (below 100 ng/ml) compared to memCD14(+) cells, although at higher concentrations (100–1000 ng/ml) the release of IL-8 was equally elevated for both memCD14(+) and memCD14(–) cells (Figure 6A). DLAM-*diP* **5** showed the highest activity in both memCD14(+) and memCD14(–) cells. Thus, the release of IL-8 induced by DLAM-*diP* appears to be memCD14-dependent at the low concentration range (0.1–100 ng/ml), whereas at concentrations of 100 ng/ml and more, the signaling induced by DLAM-*diP* was not further enhanced by presence of memCD14. In contrast, the production of IL-8 induced by DLAM-*monoP* **3**, **6**, and **7** was largely memCD14-dependent in the whole concentration range revealing DLAM-*monoP* **6** (the monophosphate counterpart of DLAM-*diP* **5**) as the most efficient (Figure 6B). In general, DLAMs-*diP* **5** having shorter secondary acyl chain at position 6 (presented at the secondary dimerization interface) showed superior activity, whereas DLAM-*diP* **1** having longer secondary acyl chain at position 4 was the least active.

### αα-GM-DLAMs Induce NF-κB Signaling in mTLR4/mMD-2 Transfected HEK293 Cells

DLAMs induced dose-dependent graded activation of NF-κB–regulated signal transduction pathway in mTLR4/mMD-2 transfected HEK293 cells (Figure 7). The mTLR4 activation profiles induced by DLAMs belonging to the same structural series were similar and the 4-lipidated-series (DLAMs **1** and **2**) was less active compared to the 6-lipidated series (DLAMs **4** and **5**) (Figure 7A). Thus, murine TLR4 discriminated the sites of acylation and phosphorylation at the proximal sugar (Mannose which presumably faces the secondary dimerization interface) but was unresponsive to the length of secondary acyl chain (C_10_ or C_12_) that is, most likely, exposed at the dimerization interface. Along these lines, the spatial arrangement of lipid chains at the dimerization interface induced pronounced effect on the mTLR4-mediated activity. As expected, the monophosphates **3**, **6**, and **7** were less efficient in inducing NF-κB activation compared to *E. coli Re*-LPS, although DLAMs-*monoP* **3** and **6** overperformed the MPLA. The activation profiles induced by 4-lipidated DLAM diphosphates **1** and **2** (Figure 7A) were comparable to those induced by DLAMs-*monoP* **3** and **6** (Figure 7B). Thus, ionic contacts play a relatively minor role in the homodimerization process of mTLR4/mMD-2/DLAM complexes and the dimerization is driven majorly by hydrophobic interactions in this case. Indeed, DLAM-*mono*P **3**, a monophosphate counterpart of DLAM-*di*P **1**, was only 3-fold less active in inducing mTLR4-driven NF-κB activation compared to its diphosphate counterpart, whereas in hTLR4/HEK293 cells DLAM **3** was 70-fold less active compared to DLAM **1** (Figure 5).

### Picomolar Concentrations of αα-GM-DLAMs Induce Dose-Dependent Release of TNF-α and IL-6 in Mononuclear Cells (MNC)

To test the impact of DLAMs diphosphates **1, 2**, and **4, 5** and DLAMs monophosphates **3, 6**, and **7** on the induction of pro-inflammatory signaling in primary immune cells, the release of IL-6 and TNF-α in human MNC was examined (Figure 8). The expression of IL-6 induced by picomolar concentrations of DLAMs diphosphates (αα-GM-DLAMs-*diP*) could be gradually modulated by altering the chemical structure of the molecules, i.e., by varying the position of acyl chains and the phosphate group at the Man moiety. Comparison of compounds belonging to different structural series (but possessing the lipid chains of equal length) revealed the 6-lipidated 4-phosphates **4** and **5** more efficient in producing IL-6 than the corresponding 4-lipidated 6-phosphates **1** and **2**, respectively (compounds possessing lipid chains of equal length are compared) (Figure 8A). Shortening secondary acyl chain (C_10_ vs. C_12_) resulted in substantial enhancement of the IL-6 release: compounds **2** and **5** (having C_10_ secondary lipid chain facing secondary dimerization interface) were more efficient in inducing the expression of IL-6 than the DLAMs **1** and **4** with longer secondary lipid chain (C_12_) (compounds having equal phosphorylation pattern are compared). A similar tendency was observed for the DLAM-induced TNF-α release (Figure 8B). Examining MNC stimulation by higher concentrations of DLAMs (Figures 8C,D) revealed that the release of IL-6 and TNF-α reaches a plateau at the concentrations higher than 500 pg/ml of TLR4 agonist (saturation of all expressed TLR4 complexes combined with possible CD14-dependent endocytosis), whereas the highest level of cytokine release depends on the chemical structure of DLAM. DLAM **1** induced expression of the lowest level of TNF-α, although it was efficient in inducing the release of IL-6, whereas DLAM **2** showed a reversed profile. DLAM **5** demonstrated the highest potency in triggering the expression of both TNF-α and IL-6. In general, the structure-activity relationships in MNC followed the same pattern as in all other cell lines tested: 6-lipidated DLAMs were more active than their 4-lipidated counterparts, and shortening of secondary acyl chain exposed at the dimerization interface (position 4 or 6 of Man) resulted in the elevation of TLR4 stimulating potential. Along this lines, synthetic manipulation of the length of secondary acyl chain and the sites of lipidation in DLAMs can be used for fine-tuning of the TLR4-mediated cytokine release.

Dampened induction of TNF-α and IL-6 by DLAMs monophosphates (αα-GM-DLAMs-*monoP*) correlated with their chemical structure missing a phosphate group at the Man moiety which confirmed the importance of ionic interactions at the dimerization interface (Figures 8E,F) (50). The modulation of cytokine production induced by αα-GM-DLAMs-*monoP* could be achieved through varying the position and the length of lipid chains at Man moiety. Thus, 6-acyloxyacyl DLAM **6** revealed the highest potency in inducing IL-6 and was somewhat more active than MPLA and 4-acyloxyacyl DLAMs **3** and 4,6-bis-lipidated DLAM **7**.

To assess the binding affinity of DLAMs diphosphates having variable acylation pattern, we performed competition experiments by simultaneous challenge of MNC with αα-GM-DLAMs-*diP* and synthetic TLR4 antagonist DA193 (38, 45) (Figure 9). Upon stimulation with 500 pg/mL of αα-GM-DLAMs-*diP* in the presence of increasing concentration of DA193, the DLAM **5** revealed the highest binding affinity (higher concentrations of DA193 were needed to suppress TNF-α and IL-6 release induced by DLAM **5**). DLAM **2** revealed somewhat lower affinity compared to DLAM **5**, whereas the cytokine release induced by DLAMs **1** and **4** could be inhibited by significantly lower doses of DA193 (Figure 9A). Application of increasing concentration of αα-GM-DLAMs-*diP* in the presence of 50 ng/mL DA193 revealed comparable results clearly highlighting DLAM **5** as most efficient in dimerizing the hTLR4/MD-2 complex, followed by DLAMs **2** and **4** (Figure 9B).

### αα-GM-DLAMs Induce Cytokine Release in TPA-Primed THP-1 Macrophages in a Dose-Dependent Manner

Picomolar concentrations of αα-GM-DLAMs-*diP* **1, 2, 4**, and **5** induced the expression of TNF-α, IL-1β, and monocyte chemotactic protein-1 (MCP-1) in the TPA-primed THP-1 cells (Figure 10). DLAMs **1** and **4** having longer secondary acyl chains in position 4 or 6 of Man moiety, respectively, displayed similar dose-dependent activation profiles, although 6-phosphate **1** was less efficient compared to 4-phosphate **4** in respect to release of TNF-α and MCP-1 (Figures 10A,B), and the potencies in induction of IL-1β were comparable (Figure 10C). DLAMs **2** and **5** having shorter secondary acyl chains were generally more potent in inducing the release of TNF-α and MCP-1, but somewhat less efficient in release of IL-1β compared to their longer-chain counterparts **1** and **4**.

### DLAMs Induce the Production of IL-6 and IL-8 in Bronchoepithelial Cell Lines

**Figure 11:**
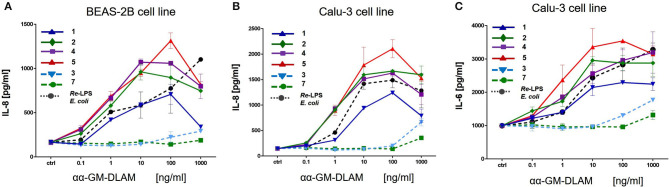
Expression of IL-8 and IL-6 induced by αα-GM-DLAMs in human epithelial cell lines compared to *E. coli* O111 LPS. **(A)** Expression of IL-8 in human bronchial epithelial cell line BEAS-2B; **(B)** Expression of IL-8 in cultured human airway epithelial cell line Calu-3; **(C)** Expression of IL-6 in cultured human airway epithelial cell line Calu-3. Data were combined from *n* = 2 independent experiments, error bars indicate standard error of the mean.

αα-GM-DLAMs-*diP* induced dose-dependent release of IL-8 and IL-6 in cultured human airway epithelial cell lines Calu-3 and BEAS-2B, whereas 6-lipidated 4-phosphorylated DLAM **5** was the most potent and its 4-lipidated 6-phosphorylated counterpart DLAM **1** was the least active in induction the expression of IL-6/IL-8 (Figure 11). Interestingly, at lower concentrations (0.1–100 ng/mL) DLAMs **2, 4**, and **5** overplayed *E. coli Re*-LPS in the expression of IL-6/IL-8 whereas at higher concentrations (100–1000 ng/mL) the inflammatory responses induced by DLAMs declined. The monophosphates αα-GM-DLAMs-*monoP* showed very low activating potential in epithelial cell lines which is related to the CD14 dependency of DLAMs-*mono*P (discussed earlier). CD14 is poorly expressed by cultured human airway epithelial cells which has apparently a pronounced impact on the sensitivity of this cell lines to the monophosphorylated DLAMs. Besides, DLAMs had to be applied in nM doses to induce cytokine release in bronchoepithelial cell lines which was attributed to the lower expression level of MD-2 in human airway epithelia (51).

### Tailored Induction of TNF-α and IL-6 in mBMDMs by Structurally Different DLAMs

**Figure 12:**
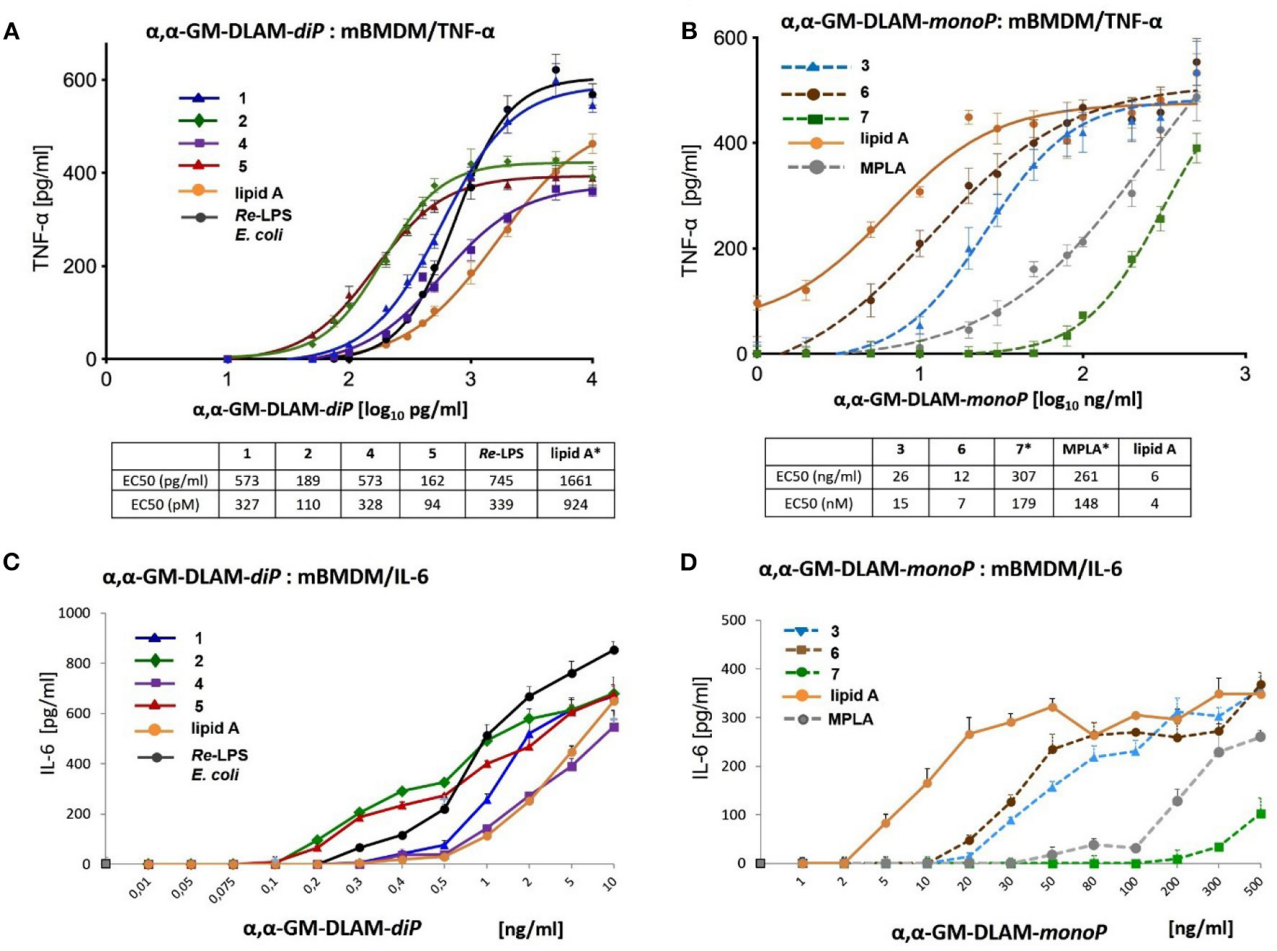
Dose-dependent expression of cytokines in mouse BMDM induced by αα-GM-DLAMs. **(A)** Expression of TNF-α induced by αα-GM-DLAM-*diP* **1, 2, 4**, and **5** compared to *E. coli Re*-LPS and synthetic *E. coli* lipid A; **(B)** expression of TNF-α induced by αα-GM-DLAM-*monoP* **3, 6**, and **7** compared to synthetic *E. coli* MPLA and synthetic *E. coli* lipid A. Non-linear curve fit (variable slope, four parameters) was calculated using GraphPad Prism. EC50 in [pM] were calculated based on the following MW: DLAM 1 = 1751 Da, DLAM 2 = 1723 Da, DLAM 4 = 1751 Da, Fa148 = 1723 Da, *E. coli Re*-LPS = 2200 Da, DLAM 7 = 1715 Da, DLAM 3 = 1671 Da and DLAM 6 = 1671 Da; *EC50 calculation is subject to increased uncertainty due to limited data points at maximal response. **(C)** expression of IL-6 induced by αα-GM-DLAM-*diP* **1, 2, 4**, and **5** compared to *E. coli Re*-LPS and synthetic *E. coli* lipid A; **(D)** expression of IL-6 induced by αα-GM-DLAM-*monoP* **3, 6**, and **7** compared to synthetic *E. coli* MPLA and synthetic *E. coli* lipid A. Data were combined from *n* = 3 independent experiments; error bars indicate standard error of the mean. GraphPad Prism 9 was used to perform statistical analysis.

**Figure 13:**
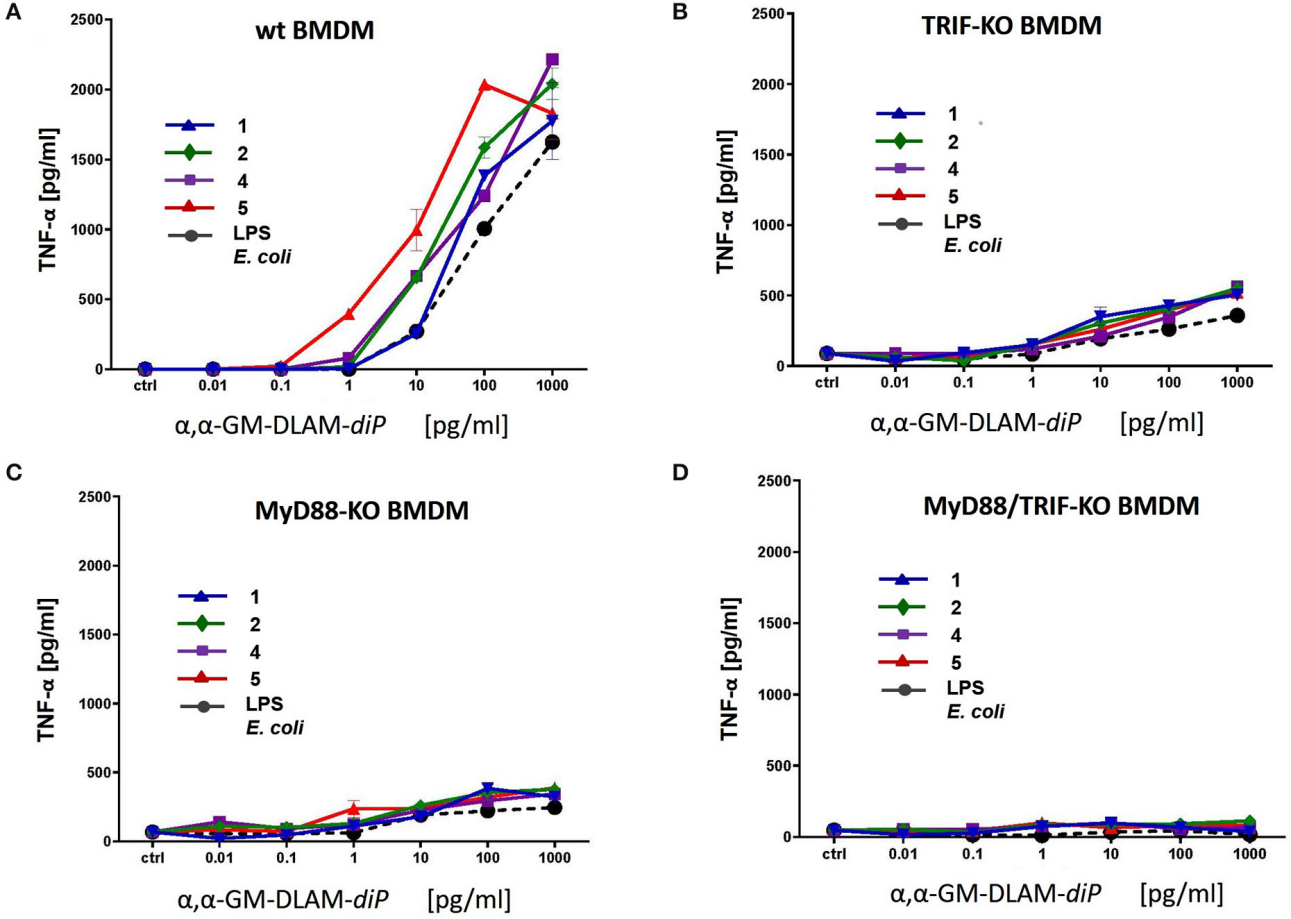
Release of TNF-α in immortalized mouse macrophages induced by increasing concentrations of αα-GM-DLAM-*diP* **1, 2, 4**, and **5**. Cell lines were generated from **(A)** wt; **(B)** TRIF-KO; **(C)** MyD88-KO; **(D)** MyD88/TRIF-KO bone-marrow-derived macrophages. Data shown are exemplary for *n* = 2 independent experiments, error bars indicate standard deviation of the mean.

Next, we examined cytokine profiles induced by increasing concentrations of DLAMs in mBMDMs compared to *E. coli Re*-LPS, MPLA, and synthetic *E. coli* lipid A (Figure 12). The monitored responsiveness of native murine immune cells to DLAMs was significantly higher compared to the mTLR4 transfected HEK293 cells (picomolar vs. nanomolar range, respectively). Similar to data obtained for human system, DLAMs **2** and **5** was the most powerful inducer of TNF-α and IL-6 release, whereas DLAMs **1** and **4** where the secondary acyl chain was lengthened by 2xCH_2_ atoms (C_12_) were less efficient (Figures 12A,C). All four DLAMs-*di*P were more effective in induction of TNF-α and IL-8 release than synthetic *E. coli* lipid A. Evaluation of cytokine-inducing potency of DLAMs monophosphates highlighted the 6-lipidated DLAM-*mono*P **6** as the most efficient (which is also fully consistent with results obtained in human MNC), followed by 4-lipidated DLAM-*mono*P **3** and **7** (Figures 12B,D). Obviously, DLAM-*mono*P **3** and **6** overperformed MPLA in induction of both TNF-α and IL-6. Thus, comparative potency of structurally different DLAMs to induce cytokine release in murine primary immune cells nicely correlated with the data obtained in human primary immune cells providing a proof for species unrelated structure-activity relationships. In order to corroborate that all DLAMs still follow the same rules with respect to MyD88- and TRIF-dependency as *E. coli*-LPS does, all lipid A mimetics were tested in immortalized mouse macrophage cell lines, deficient for MyD88, TRIF, or both. As expected, both individual knockouts showed drastically reduced but still measurable induction of TNF-α, whereas the double KO cell line did not show any induction at all (Figure 13).

## Discussion

**Figure 14:**
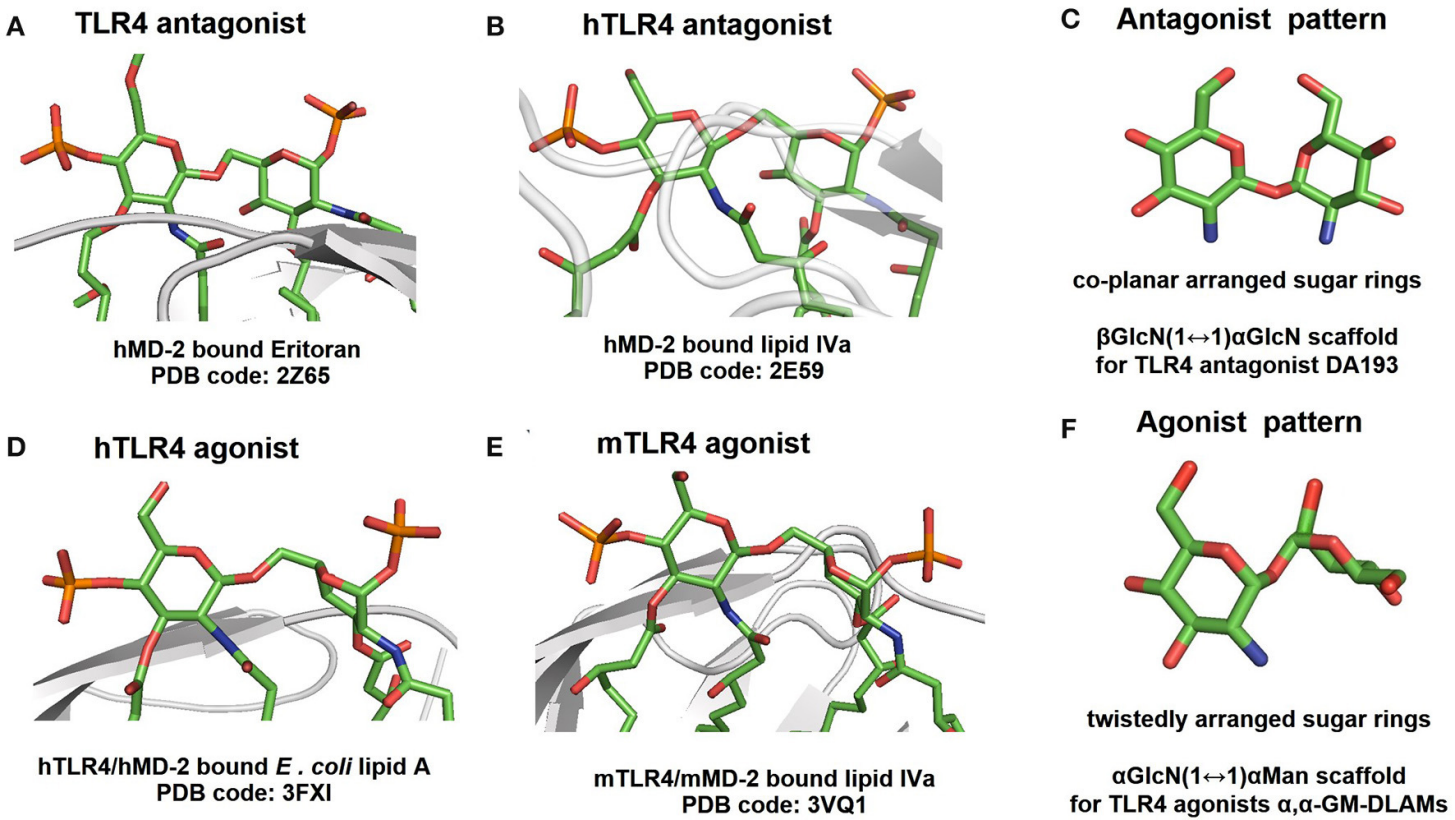
3D-tertiary structure of the diglucosamine backbone of protein-bound lipid A variants taken from the co-crystal structures of: **(A)** hMD-2 bound TLR4 antagonist Eritoran; **(B)** hMD-2 bound TLR4 antagonist lipid IVa; **(C)** 3D molecular shape of β, α-(1↔1)-linked diglucosamine as scaffold for synthetic TLR4 antagonists; **(D)** hTLR4/MD-2 bound TLR4 agonist *E. coli* lipid A (only lipid A portion of *Ra*-LPS is shown for clarity); **(E)** mTLR4/MD-2 bound TLR4 agonist lipid IVa. **(F)** 3D molecular shape of α,α-(1↔1)-linked disaccharide as scaffold for synthetic TLR4 agonists. Note for **(A,B,D,E)**: the distal GlcN ring is fixed in the same orientation for all structures, the two GlcN rings are co-planar arranged in **(A,B)**, whereas in **(D,E)** the proximal GlcN ring is in twisted orientation.

The affinity of lipid A to MD-2 is largely determined by its primary chemical structure and depends on the number, lengths and structure of lipid chains and the phosphorylation status of its β(1 → 6)-linked diglucosamine backbone (52, 53). Likewise, the efficiency and tightness of ligand-induced TLR4 complex dimerization is governed by the acylation pattern of lipid A and the presence/absence of negative charges (e.g., phosphate groups). Since the 3D-conformation of the diglucosamine backbone of lipid A can be easily amended by rotation about highly flexible β(1 → 6) glycosidic linkage (54), the disaccharide backbone of lipid A readily changes its molecular shape upon binding by the MD-2/TLR4 complex. Consequently, the GlcN rings of the diglucosamine backbone adopt a co-planar relative arrangement for MD-2/TLR4-bound antagonist lipid A variants (Figures 14A,B) and a skewed relative arrangement for MD-2—bound agonist ligands (Figures 14D,E). We have recently shown that the 3D tertiary structure of the MD-2—bound lipid A is decisive for the expression of specific biological activity and that the particular binding orientation of the lipid A molecule in the binding grove of MD-2 (±180°) can be impelled by fixing the 3D-molecular shape of the disaccharide skeleton of lipid A in a pre-defined conformation (41, 45). Following this concept, we have developed potent synthetic TLR4 antagonists derived from a co-planar arranged β,α-(1↔1)-linked diglucosamine scaffold (Figure 14C) (38, 55) and TLR4 agonists based on a twistedly shaped disaccharide scaffold (Figure 14F, this paper) (41). Notably, our approach allows to surmount species-specificity in ligand recognition by the TLR4 complex for all types of biological activities (endotoxic/antiendotoxic) and to predictably and gradually modulate TLR4 activation.

Thus, in contrast to native lipid A derived from a flexible three-bond linked βGlcN(1 → 6)GlcN backbone which can spontaneously adjust its molecular shape upon binding to the MD-2/TLR4 complex (Figures 2D, 3A), the TLR4 agonist DLAMs are based on the extraordinary rigid α,α-1,1′-linked spaciously twisted disaccharide backbone mirroring the molecular shape of the MD-2 bound *E. coli* lipid A. The relative orientation of two sugar rings in the α,α-(1↔1) linked disaccharides depends on the conformation of glycosidic linkage (more precisely, on the torsion angles around glycosidic (1↔1) linkage) and is governed by specific carbohydrate related effects (the anomeric and exo-anomeric effects). The unique “skewed” 3D-molecular shape of α,α-(1↔1) linked disaccharides was determined by X-ray crystallography: in particular, it was demonstrated that the conformation of α,α-(1↔1) linkage is conserved in all circumstances and that the characteristic “skewed” molecular shape of α,α-(1↔1) linked disaccharides is sustainably maintained and does not depend on the nature of functional groups attached at either sugar ring (56–59). The exceptional rigidity of an α,α-(1↔1) glycosidic linkage and a restrained skewed arrangement of α,α-(1↔1)-linked sugar rings was also confirmed by molecular dynamics simulations that corroborated the existence of a single conformational minimum (54, 60–62). Thus, conformationally restrained αGlcN(1↔1)αMan disaccharide with its twistedly oriented sugar rings mirroring the 3D-tertiary structure of the diglucosamine skeleton of MD-2/TLR4–bound *E. coli* lipid A provides a versatile scaffold for synthetic TLR4—activating lipid A mimetics.

Initially we assessed the specificity of synthetic lipid A mimetics toward human and mouse TLR4/MD-2 complexes by examining the ability of DLAMs to induce the pro-inflammatory signaling in hTLR4/hMD-2/CD14(±) and mTLR4/MD-2 transfected human embryonic kidney (HEK)293 cells, respectively. DLAMs **1** and **4** having 2 × CH_2_ longer acyl side chain at the Man residue facing secondary dimerization interface were less efficient activators of NF-κB compared to DLAMs **2** and **5**. Thus, shortening the secondary acyl chain by 2 × CH_2_ resulted in enhanced TLR4-mediated signaling, whereas the sites of attachment of acyl chains and phosphate group (position 4 or 6) did not exert significant effect on the efficiency of SEAP induction (Figure 5).

We also examined the memCD14-dependency of DLAMs by comparing the IL-8 release induced by DLAMs in HEK293 cells transfected with hTLR4/hMD-2/hCD14 or hTLR4/hMD-2 (Figure 6). memCD14 enhanced the sensitivity of hTLR4/MD-2 complex to DLAMs diphosphates at picomolar concentration range (0.1–10 ng/mL) (Figure 6A). At higher concentrations (100–1000 ng/mL) the hTLR4 activation induced by DLAMs was not significantly influenced by the presence of CD14. In contrast, DLAMs monophosphates, similarly to MPLA, were CD14—dependent in the whole concentration range showing no activity below 1000 ng/ml in the CD14 deficient cells. Since DLAM-*monoP* **3** is a 6-dephosphorylated counterpart of the DLAM-*diP* **1** and DLAM-*monoP* **6** is a 4-dephosphorylated counterpart of the DLAM-*diP* **4**, and both DLAMs-*monoP* are “mimetics” of MPLA, the CD14—dependency is closely related to the presence of the second phosphate group (phosphate group exposed at the secondary dimerization interface) in the molecule. CD14 is known to enhance the sensitivity of the TLR4 complex to endotoxin by transferring LPS from LBP to the binding pocket of MD-2 (48, 63), as well as to be involved in the CD14—dependent TLR4/MD-2/LPS endocytosis (64, 65) and in the regulation of tightness of the TLR4 complex dimerization (66).

Monophosphorylated DLAMs, as expected, showed reduced activity compared with DLAMs diphosphates, but still overperformed MPLA in inducing NF-κB regulated signal transduction pathway both at h- and mTLR4. For DLAMs-*monoP* the TLR4 activation profiles were similar in human and mouse systems highlighting DLAM **6** as the most potent, followed by DLAMs **3** and **7** (Figures 6B, 7). Thus, modulation the activity of DLAMs-*monoP* was achieved by varying acylation sites at the Man moiety (acyloxyacyl chain at position 6 for DLAM **6** and at position 4 for DLAM **3**) and two 4,6-hydroxyacyl chains for DLAM **7**.

To examine the impact of αα-GM-DLAMs on cytokine induction in MNC, mononuclear cells were stimulated with a wide concentration range of DLAMs or with *E. coli* LPS and MPLA as controls. Picomolar doses of DLAMs-*di*P were sufficient to induce potent induction of TNF-α and IL-6 in human MNC (Figures 8A,B). Dose-dependent release of IL-6 and TNF-α induced by higher concentrations of DLAMs allowed for determination of EC50 values which were in the picomolar range for DLAMs diphosphates (Figures 8C,D). The induction of cytokines by αα-GM-DLAMs correlated to their chemical structure revealing shorter-chain 6-lipidated DLAM **5** as the most potent in inducing the release of TNF-α and IL-6, followed by shorter-chain 4-lipidated DLAM **2**, whereas DLAMs **4** and **1** having longer secondary acyl chain at Man (secondary dimerization interface) were somewhat less efficient in the picomolar range (below 500 pg/ml). At higher concentrations, the maximum responses induced by DLAMs-*diP* differed for release of IL-6 and TNF-α disclosing DLAMs **4** and **5** as most efficient in respect to IL-6 secretion, whereas the DLAMs **5** and **2** revealed higher potency (lower EC50 values). Accordingly, synthetic manipulations of the length of acyl side-chain as well as the site of attachment of acyloxyacyl residue and the phosphate group at Man moiety allowed for the fine-tuning of the TLR4-mediated cytokine release.

The cytokine-inducing potential of DLAMs-*mono*P was associated with their primary chemical structure missing a phosphate group at the dimerization interface and dropped from picomolar to nanomolar concentration range. The structure-activity relationships among DLAMs-*monoP* were cell-line and species-independent and revealed the 6-acyloxyacyl DLAM **6** (a monophosphorylated counterpart of DLAM **4**) as the most potent, followed by 4-acyloxyacyl DLAM **3** (a monophosphorylated counterpart of DLAM **1**) and 4,6-lipidated DLAM **7** (Figures 8E,D). Notably, DLAM **6** overplayed *S. minnesota* MPLA in inducing the release of both TNF-α and IL-6 in MNC.

Remarkably, *E. coli Re*-LPS (Kdo_2_-lipid A)—which has at least a 10-fold higher potency in TLR4-mediated activation of cytokine release than the corresponding lipid A (67)—was less powerful in inducing TNF-α and MCP-1 in TPA-primed THP-1 macrophages compared to DLAMs-*di*P (Figures 10A,B). Two Kdo residues attached at position 6 of lipid A (*Re*-LPS) are sufficient to endorse lipid A with a full *E. coli* LPS-like activity (68). Also the presence of only one Kdo residue is known to substantially enhance the TLR4-dependent activity of Kdo-lipid A compared to lipid A alone (35). The induction of NF-κB signal transduction pathway by diphosphate DLAMs-*di*P was as strong as the response elicited by *E. coli Re*-LPS indicating that the unique structural features of Kdo-deficient DLAMs render lipid A mimetics exceptionally powerful TLR4 agonists. This has been confirmed also in mouse BMDMs where DLAMs **2** and **5** showed 10-fold higher potency in inducing TNF-α compared to *E. coli* lipid A and were also more potent than *Re*-LPS (Figure 12A). Monitoring the secretion of IL-1β in TPA-primed THP-1 macrophages revealed an inverted activation profile, with longer-chain αα-GM-DLAM **1** being most active, indicating the involvement of different structural factors in ligand recognition for induction of IL-1β pathway (Figure 10C) (69). Indeed, intracellular LPS-mediated IL-1 release in human monocytic cell lines is dependent on caspase-4/5 which regulates the secretion of inflammasome-activated IL-1β (70, 71). Activation of the NLRP3 inflammasome in macrophages requires two simultaneous signals: the LPS-induced activation of the NF-κB pathway as a priming stimulus which activates the transcription of NLRP3 and pro-IL-1β, as well as a second signal to induce the assembly of inflammasome complex via recruitment of pro-caspase-1 and adaptor molecule ASC (apoptosis-associated speck-like protein containing a caspase-recruitment domain) (72, 73). Although macrophages are believed not being able to release IL-1β in response to LPS without exogeneous ATP as 2nd stimulus (74), it has been demonstrated before that differentiation of THP-1 cells using high concentrations of TPA (100–200 nM) or prolonged differentiation times induces a more activated macrophage phenotype (75, 76). Such macrophage phenotype is associated with secretion of IL-1β in response to LPS alone as a result of spontaneous release of endogenous ATP and a moderate activation of caspase-1 (77). Similar mechanisms may be involved in our experimental set-up as well. Additionally, CD14-mediated internalization of TLR4/MD-2/LPS complexes into endosomal compartments in TPA-primed THP-1 macrophages by a process mediated by tyrosine kinase Syk and its downstream effector phospholipase C (PLC) γ2 could follow the LPS or DLAMs challenge (64, 65). Therefore, we suppose that the DLAM-mediated caspase-4/5 activation in TPA-primed THP-1 cells culminating in elevated IL-1β secretion may occur by a physiological process of DLAM-induced CD14—dependent TLR4/MD-2/DLAM internalization and activation of cytosolic LPS-sensing receptors. The specificity and high affinity of DLAMs-*di*P to CARD of caspase-4 has been previously confirmed in *in-vitro* studies (44).

We also assessed the cytokine-inducing capacity of DLAMs in two human bronchoepithelial cell lines, BEAS-2B and Calu-3. The immune response of lung epithelium toward LPS has gained a lot of interest over the last years, in particular with respect to allergy and asthma (78). Tailored activation of immune signaling in lung epithelium through lipid A-like compounds may therefore provide possible therapeutic interventions for skewing the early immune response away from Th2 bias. The responsiveness of human airway epithelial cells toward LPS is limited due to low expression of MD-2, Jia et al. (51) therefore, nanomolar concentrations of DLAMs were required to induce cytokine release in bronchoepithelial cells. Regarding secretion of IL-8, lipid A mimetics behaved similarly in both cell lines revealing DLAMs **5** and **4** the most potent inducers of pro-inflammatory signaling. Longer chain 4-lipidated DLAM **1** showed substantially diminished IL-8 release. At higher DLAMs concentrations (100–1000 ng/mL) the activity abruptly dropped which could be related to a change in the aggregation state of DLAMs in aqueous solutions requiring the action of mem-CD14 for efficient transfer of a single DLAM molecule to MD-2. Membrane-bound CD-14 that augments cellular response to LPS is not sufficiently expressed in bronchoepithelial cell lines (79) which results in poorer activity of CD14-dependent TLR4 ligands. Along these lines, the monophosphate DLAMs **3** and **7** were nearly inactive at the concentrations tested, which is consistent with the full CD14 dependency of DLAMs-*monoP*-induced TLR4 activation. An almost identical pattern of agonist activity was monitored for the induction of IL-6 release with DLAM **5** as the most potent and DLAM **1** as the least efficient.

Generally, we assessed two essential properties of DLAMs to interact with TLR4 complex, namely, affinity and efficacy. The affinity of DLAMs to TLR4/MD-2 complex can be defined as the ability to bind to MD-2, whereas efficacy can be determined as an inherent property of the MD-2-bound DLAM to induce the dimerization and activation of the TLR4 complex. Along these lines, the EC50 values could describe the affinity of DLAMs to MD-2/TLR4 complex, whereas efficacy is more related to maximum response. Different DLAMs can bind to MD-2/TLR4 with similar affinity but differ in terms of relative efficacy (maximum response): for example, DLAMs **1** and **4** or DLAMs **2** and **5** in BMDMs (Figure 12A) or DLAMs **2** and **5** in MNC (Figures 8C,D). We measured the activity by secretion of cytokines (that may also lead to auto-self-activation) as well as by other parameters which by themselves can be influenced by additional factors. Hence, the whole test system for assessment of TLR4 ligands affinity, activity, and potency is very complex and that was the reason for using many different *in-vitro* systems to examine the potency of DLAMs to activate TLR4-mediated signaling. Since the recognition of LPS or DLAMs is a multifaceted multistep process where many innate immune proteins are involved, specific structural features of DLAMs could have enormous impact on the mode of their binding to both CD14 and MD-2. This can also affect the tightness and the mode of the TLR4 complex dimerization which, in turn, could result in differential cytokine release. CD14 has been shown to bind a single LPS molecule (49) and shuttle it to the binding pocket of MD-2 (48), although the atomic structure of LPS/CD14 complex has not yet been solved and the structural basis of LPS binding by CD14 is largely unknown. We suppose that the mode of interaction of DLAM with CD14 might also have significant impact on the way how DLAMs are “inserted” into the binding pocket of MD-2 and, consequently, on the following signaling events. Also, the DLAM “load” must have substantial effect on the recognition process since cellular responses to low concentrations of DLAMs are fully memCD14 dependent, whereas the induction of the pro-inflammatory signaling at higher concentrations of DLAMs can proceed in memCD14 independent manner.

Due to their specific structural features such as rigidified disaccharide backbone mimicking the molecular shape of MD-2-bound lipid A, acylation pattern mirroring the one of *E. coli* LPS, homogeneity, stability and defined chemical structure, DLAMs represent versatile tools for studying the structural basis of lipid A recognition by proteins of the LPS transfer cascade and the molecular basis of TLR4 activation.

## Concluding Remarks

In addition to primary chemical structure, we manipulated the three-dimensional molecular shape of lipid A and designed a novel class of TLR4/MD-2-specific glycolipids based on the conformationally restricted synthetic α,α-(1↔1)-linked disaccharide scaffold reflecting the 3D-tertiary structure of TLR4/MD-2-bound *E. coli* lipid A. These specific structural features of DLAMs, that were created using approaches of synthetic glycochemistry, ensured picomolar affinity to TLR4/MD-2, species-independency in respect to human and mouse systems, and the possibility to predictably modulate the TLR4-mediated activity by chemical modifications. The TLR4/MD-2-mediated cellular pro-inflammatory responses were induced by αα-DLAMs in a CD-14 dependent manner and could be readily modulated through modification of the primary chemical structure of synthetic glycolipids. Both the position of phosphate group (at C-4 or C-6 of Man) facing secondary dimerization interface and the length of acyl chains were accountable for modulation of human and murine TLR4—stimulating activity. DLAMs having shorter secondary acyl chain (C_10_) were generally more active than DLAMs having longer secondary acyl chain (C_12_). As to phosphorylation pattern, the 4-phosphate DLAMs (lipidated at position 6 of Man) were more potent than the corresponding 6-phosphates (lipidated at position 4 of Man; compounds having lipid chains of equal length are compared). DLAMs lacking the phosphate group at the sugar moiety closest to the secondary dimerization interface (monophosphate DLAMs) induced diminished inflammatory responses and performed as weak TLR4 agonists showing similar tendency toward higher activity of 6-*O*-lipidated counterpart. For tailored modulation of TLR4-mediated signaling induced by DLAMs, the positioning of phosphate groups and acyloxyacyl residues decorating the disaccharide backbone, as well as the length of lipid chains can be manipulated by chemical modifications. The chemical synthesis would allow to create an even more broad diversity of conformationally restricted DLAMs varying in the sites of acylation and phosphorylation as well as in the length of acyl chain bordering secondary dimerization interface (C_6_ to C_16_ in length) to warrant customized modulation of TLR4-mediated cellular responses. All these features render DLAMs promising candidates for further development into TLR4-dependent immunomodulating therapeutics which can enhance the innate immune responses as well as into potential vaccine adjuvants.

## Data Availability Statement

The original contributions presented in the study are included in the article/supplementary material, further inquiries can be directed to the corresponding author/s.

## Author Contributions

AZ conceived the project. AZ, HH, RB, and RJ analyzed literature and designed detailed research. All authors performed their respective experiments and interpreted results. AZ, HH, RB, SI, and TP analyzed the data and wrote the manuscript. All authors read and approved the final manuscript.

## Conflict of Interest

The authors declare that the research was conducted in the absence of any commercial or financial relationships that could be construed as a potential conflict of interest.
